# Autophosphorylation of conserved yeast and human casein kinase 1 isozymes regulates Elongator-dependent tRNA modifications

**DOI:** 10.1093/nar/gkaf881

**Published:** 2025-09-10

**Authors:** Maria Friederike Landrock, Rościsław Krutyhołowa, Pauline Böhnert, Jarosław Mazur, Małgorzata Honc, Alexander Hammermeister, Larissa Bessler, David Scherf, Anna Elms, Natalia Radczuk, Bozena Skupien-Rabian, Urszula Jankowska, Friedrich W Herberg, Mark Helm, Roland Klassen, Sebastian Glatt, Raffael Schaffrath

**Affiliations:** Department of Microbiology, Institute of Biology, University of Kassel, 34132 Kassel, Germany; Małopolska Centre of Biotechnology, Jagiellonian University, 30387 Krakow, Poland; Institute of Molecular Biology and Biophysics, ETH Zurich, 8093 Zurich, Switzerland; Department of Microbiology, Institute of Biology, University of Kassel, 34132 Kassel, Germany; Małopolska Centre of Biotechnology, Jagiellonian University, 30387 Krakow, Poland; Institute of Medical Microbiology, University of Zurich, 8006 Zurich, Switzerland; Małopolska Centre of Biotechnology, Jagiellonian University, 30387 Krakow, Poland; Department of Microbiology, Institute of Biology, University of Kassel, 34132 Kassel, Germany; Małopolska Centre of Biotechnology, Jagiellonian University, 30387 Krakow, Poland; Institute of Pharmaceutical and Biomedical Sciences, Johannes Gutenberg University of Mainz, 55128 Mainz, Germany; Department of Microbiology, Institute of Biology, University of Kassel, 34132 Kassel, Germany; Department of Microbiology, Institute of Biology, University of Kassel, 34132 Kassel, Germany; Małopolska Centre of Biotechnology, Jagiellonian University, 30387 Krakow, Poland; Małopolska Centre of Biotechnology, Jagiellonian University, 30387 Krakow, Poland; Małopolska Centre of Biotechnology, Jagiellonian University, 30387 Krakow, Poland; Institute of Biology, Department of Biochemistry, University of Kassel, 34132 Kassel, Germany; Institute of Pharmaceutical and Biomedical Sciences, Johannes Gutenberg University of Mainz, 55128 Mainz, Germany; Department of Microbiology, Institute of Biology, University of Kassel, 34132 Kassel, Germany; Małopolska Centre of Biotechnology, Jagiellonian University, 30387 Krakow, Poland; Department for Biological Sciences and Pathobiology, University of Veterinary Medicine Vienna, 1210 Vienna, Austria; Department of Microbiology, Institute of Biology, University of Kassel, 34132 Kassel, Germany

## Abstract

Casein kinase 1 (CK1) family members are crucial for ER-Golgi trafficking, calcium signalling, DNA repair, transfer RNA (tRNA) modifications, and circadian rhythmicity. Whether and how substrate interactions and kinase autophosphorylation contribute to CK1 plasticity remains largely unknown. Here, we undertake a comprehensive phylogenetic, cellular, and molecular characterization of budding yeast CK1 Hrr25 and identify human CK1 epsilon (CK1ϵ) as its ortholog. We analyse the effect of Hrr25 depletion and catalytically inactive mutants *in vivo* and show that perturbations in CK1 activity lead to stress-induced growth defects, morphological abnormalities, and loss of Elongator-dependent tRNA modification. We use purified Hrr25 protein to identify distinct autophosphorylation patterns and phospho-sites on several physiological substrates *in vitro* and find only human isozyme CK1ϵ can replace yeast Hrr25 functions essential for tRNA modification and cell proliferation *in vivo*. Furthermore, we demonstrate that human and yeast CK1 orthologs share conserved autophosphorylation sites within the kinase domains, which regulate their activities and mutually exclusive interactions with Elongator subunit Elp1 and Sit4, a phosphatase antagonist of Hrr25. Thus, autophosphorylation controls CK1 activity and regulates the tRNA modification pathway. Our data offer mechanistic insights into regulatory roles of CK1 that are conserved between yeast and human cells and reveal a complex phosphorylation network behind CK1 plasticity.

## Graphical Abstract

**Graphical Abstract:**
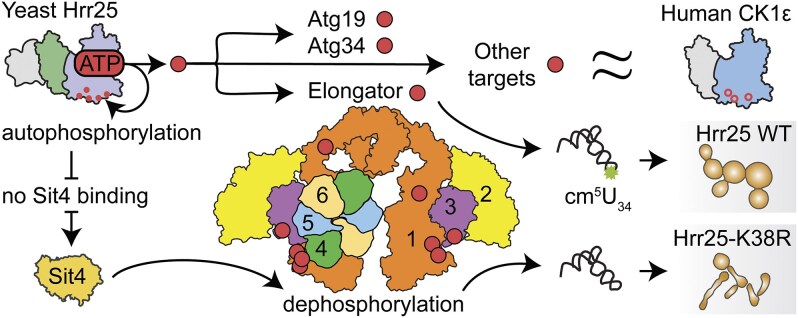

## Introduction

In their pioneering work, Burnett and Kennedy [1] performed elegant experiments with cell extracts showing that ATP can be used for phosphorylation of casein *in vitro*. Thus, the term casein kinase (CK) was coined despite the fact that casein is not a physiological substrate for phosphorylation by members of the CK families 1 and 2 (CK1 and CK2). In the human proteome, CK1 and CK2 are responsible for generating 40%–50% of all nonredundant phosphorylation sites (p-sites) [2]. Mammals harbour several functionally diverse CK1 gene subfamilies, i.e. CK1α, CK1β, CK1γ, CK1δ, or CK1ϵ [3, 4]. Hence, CK1 phosphorylation targets a wide spectrum of substrates in biological processes including Wnt signalling, autophagy and circadian rhythmicity [5–8].

Budding yeast *Saccharomyces cerevisiae* maintains one soluble CK1 isozyme (Hrr25), which is related to human CK1δ and CK1ϵ subfamilies [9, 10], and three membrane-associated versions (Yck1–Yck3) [11, 12]. The *HRR25* locus (also known as *RST2* or *KTI14*) was identified in several genetic screens, showing that yeast CK1 activity is involved in various aspects of cell biology [13–17]. For instance, Hrr25 was shown to phosphorylate various proteins related to DNA repair, ribosome biogenesis, vesicle trafficking, cell wall integrity (CWI), autophagy, calcium signalling, clathrin-mediated endocytosis, and microtubule assembly [13, 17–28]. In addition, CK1 activity is also required during meiosis, where kinase Hrr25 regulates the assembly of the meiotic chromosome axis, cohesin cleavage and monopolin function [10, 29–34]. In line with these pleiotropic roles of CK1, loss of Hrr25 and viable *HRR25* gene deletions (*hrr25*Δ) are not tolerated in haploid backgrounds of most standard yeast strains [11, 13, 15, 17]. Therefore, Hrr25 is considered essential for yeast cell viability.

Hrr25 phosphorylation has also been linked to transfer RNA (tRNA) anticodon modifications, which are catalysed by Elongator. This clinically relevant and conserved protein complex (Elp1–Elp6) is required for accurate messenger RNA translation and cell proliferation [35–43]. Intriguingly, the phosphatase Sit4/PP6 was identified as an Hrr25 antagonist in the respective tRNA modification pathway [35, 44–46]. Moreover, Sit4 and Hrr25 copurify *in vivo* [20, 47, 48] suggesting their opposing de-/phosphorylating activities are coupled to phosphoregulation of Elongator activity [46]. However, specific signals for Hrr25 recruitment to Elongator or Sit4 are unknown, and an in-depth analysis of Hrr25 phosphorylation is not available.

Here, we present comprehensive molecular analyses of Hrr25 *in vivo* and *in vitro*, including studies addressing phylogeny and functional conservation among yeast and human CK1 family members. On the basis of cross-species complementation, we show that expression of CK1ϵ, but not CK1δ, rescues phenotypes of yeast *hrr25* kinase mutants *in vivo*. In addition, we map Hrr25 p-sites in several protein substrates and CK1 itself *in vitro* and confirm the functional importance of these sites *in vivo*. Finally, we analyse differences between *cis* and *trans* autophosphorylation for the interaction of Hrr25 with Sit4 and Elongator subunit Elp1, revealing a complex phosphoregulatory network behind the tRNA modification pathway.

## Materials and methods

### Genetic manipulations in yeast and phenotypic characterization

Yeast strains used and generated in this study are enlisted in Supplementary Table S1. All strains are originated from UMY2893 [49], which contains the *SUP4* tRNA suppressor allele and the *SUP4* suppressible *ade2-1* reporter. Standard methods were used for growth and maintenance [50]. *In vivo* epitope tagging of *ELP1* with *(c-myc)_3_* was generated using a polymerase chain reaction (PCR)-based approach relying upon homologous recombination [51]. Genomic deletions targeting *ELP3* or *HRR25* were generated likewise [52]. The introduction of *in vivo* point mutations into HRR25 was accomplished through a two-step approach [53]. Confirmation of yeast genetic manipulations were made by diagnostic PCR and DNA sequencing. Oligonucleotides required for PCR are enlisted in Supplementary Table S2. For the deletion of *HRR25*, a wild-type (WT) strain (YML001) was first transformed with pCM12.2 (*HRR25*) [46] to genetically compensate the genomic deletion. Subsequently, *HRR25* was deleted by using a PCR-generated deletion cassette (*hrr25Δ:SpHIS5*), and the *URA3* plasmid pCM12.2 was eliminated by growth on synthetic complete medium containing 5-FOA (1 mg/ml). Prior to 5-fluoroorotic acid (5-FOA) chase out, plasmids encoding for *hrr25* mutants or foreign CK1 homologues from *Homo sapiens* (*HsCSNK1D*, *HsCSNK1E*) were transformed to test on ability to substitute Hrr25 function and permit cell survival. To analyse Elongator function of the different strains, growth assays were conducted as previously described [49, 54]. The role of Hrr25 on CWI was tested using calcofluor white (CFW) by addition of the compound to the media (50 μg/ml w/v) [16].

### Plasmid construction

Plasmids used and generated in this study are enlisted in Supplementary Table S3. To clone a plasmid-based WT allele of *HRR25* under endogenous promoter control, 500 bp upstream and downstream of the coding region was amplified from the yeast genome and transferred into YCplac111 or YEplac181 [55]. *hrr25* mutants were generated by shuffling the mutated sequence of pETM11-*hrr25-(1–394)* mutants into the WT *HRR25* plasmid via overlap extension PCR [56, 57] using oligonucleotides with chimeric primers (5′-ends complementary to the plasmid; 3′-end complementary to the insert). Correct integration was confirmed via DNA sequencing. Human CK1 sequences *CSNK1E*, *CSNK1D2*, and *CSNK1D3* were amplified from cDNA, originated from HEK cells. Sequences *CSNK1D1* and *CSNK1 × 1* could not be obtained from cDNA and thus, resulted from mutation of *CSNK1D2* with primers harbouring the substitution via overlap extension PCR. Moreover, the approach allowed to introduce (HA)6-tagging at C-terminus of CK1 genes [58]. Mutations of CK1 genes were carried out via PCR-based site-directed mutagenesis [59, 60] using oligonucleotides with the desired base exchanges. Tags and mutations were confirmed by DNA sequencing.

### Quantification of tRNA modifications by liquid chromatography-tandem mass spectrometry and tRNA cleavage by γ-toxin

tRNA was isolated as described by [61], hydrolysed to nucleoside level, and analysed via liquid chromatography-tandem mass spectrometry (LC-MS/MS) measurements as reported previously [62] with the sample amount being adjusted to 1 μg of digested tRNA spiked with 100 ng of internal standards (digested ^13^C-labelled tRNA from *S. cerevisiae*). For absolute quantification of biological duplicates in technical triplicates, internal and external calibration with synthetic standards was applied as detailed in [63]. Of note, the internal standard did not contain s^2^U, which is why calculations regarding s^2^U were performed with external calibration only. Finally, the total amount of modified nucleosides was normalized to the amount of uridines and related to the corresponding reference sample (set to 1). To monitor the presence of mcm^5^s^2^U_34_ modified tRNAs independent of LC-MS/MS, an *in vitro* cleavage assay using GST-γ-toxin tRNase was conducted as described [61, 64].

### Protein expression and purification

Genes encoding Hrr25 1–394 protein or its variants were cloned into pETM11 bacterial expression plasmid carrying a N-terminal 6xHis tag and TEV cleavage site. *Escherichia coli* pRARE cells were cultured in Luria broth (LB) medium until optical density (OD) 0.7, induced using 1 mM isopropyl-β-D-thiogalactopyranoside (IPTG) and incubated overnight at 18°C. GST-tagged Elp1 proteins and Sit4 were expressed from pETM30 vector in a similar fashion. For all Hrr25 purifications from bacteria, pellets were resuspended in the ice-cold lysis buffer containing 50 mM Tris, pH 7.5, 1 M NaCl, 5% v/v glycerol, 10 mM imidazole, 2 mM MgCl_2_, and 1 mM β-mercaptoethanol. In case of GST-containing Elp1 and Sit4 proteins, lysis buffer contained 300 mM NaCl. After addition of lysozyme, protease inhibitors and DNase I bacteria were sonicated on ice. To obtain soluble full length Hrr25 WT and *K38R*, corresponding genes were cloned to the pFastBacHT A carrying a N-terminal 6xHis tag and a TEV cleavage site. Proteins were expressed in the *Trichoplusia ni* High Five cells. After expression, cells were resuspended in lysis buffer and subjected to three freeze–thaw cycles in liquid nitrogen followed by sonication. Both bacterial and insect cell lysates were centrifuged at 63 000 × *g* for 45 min after sonication. Supernatants were used for NiNTA affinity chromatography using gravity flow columns. All proteins were isocratically eluted in their corresponding lysis buffers containing 250 mM imidazole. Elp1 and Sit4 proteins were further purified using GST affinity chromatography on GST-Prep FF columns (Cytiva) and eluted in lysis buffer containing 18 mM glutathione. All proteins were subjected to the gel filtration using a Superose 200 column (Cytiva) equilibrated with 20 mM Tris, pH 7.5, 500 mM NaCl, 2 mM dithiothreitol (DTT) in case of Hrr25 and 20 mM Tris, pH 7.5, 150 mM NaCl, 2 mM DTT in case of Elp1 and Sit4. Proteins in gel filtration buffer were concentrated, snap-frozen in liquid nitrogen and stored at −80°C until use.

### Phosphorylation assay

All samples of phosphorylated proteins were generated freshly prior to the experiments. To observe phosphorylation, Hrr25 proteins with or without substrates were incubated with 1 mM ATP in 20 mM Tris, pH 7.5, 150 mM NaCl, 2 mM DTT, 2 mM MgCl_2_ for 1 h at 30°C. Next, the reaction was quenched by addition of 10 mM ethylenediaminetetraacetic acid, Laemmli sample buffer was added and proteins were denatured for 5 min at 95°C. Samples were analysed on sodium dodecyl sulphate–polyacrylamide gel electrophoresis (SDS–PAGE) followed by Coomassie staining and western blotting.

### Mass spectrometry

Initial phosphorylation analyses performed for Hrr25 WT 1–394 protein were done on samples digested with the use of filter-aided sample preparation protocol [65]. The obtained peptides were directly analysed using the LC-MS/MS system described below or additionally enriched in phosphopeptides using TiO_2_ beads before the measurement. In subsequent experiments, the analysed protein mixtures were separated by SDS–PAGE and selected gel bands were further processed. To maximize a sequence coverage of analysed proteins, part of the samples were prepared in duplicates and one replicate was digested with trypsin, while the other one with chymotrypsin. At the end, results obtained for both enzymes were merged. Proteins in gel bands were washed, reduced, alkylated, and prepared for digestion as described in [66]. Then, 0.3 μg of trypsin or 0.3 μg of chymotrypsin per sample was added and digestion was performed in 25 mM NH_4_HCO_3_ (supplemented with 10 mM CaCl_2_ for chymotrypsin) overnight at 37°C or 25°C, respectively. The resulting peptides were collected, dried, and resuspended in a loading buffer (2% acetonitrile, 0.05% trifluoroacetic acid). Then, they were analysed by LC-MS/MS system consisting of Q‐Exactive mass spectrometer (Thermo Fisher Scientific) and nanoHPLC (UltiMate 3000 RSLCnano System, Thermo Fisher Scientific) using the same settings as in [66]. The acquired data were analysed with the help of Proteome Discoverer software (v.1.4; Thermo Scientific) and an in-house MASCOT search engine (v.2.5.1; Matrix Science). The SwissProt database restricted to *S. cerevisiae* taxonomy or database of common protein contaminants supplemented with sequences of studied proteins were employed to identify the content of samples. The database searching parameters were as follows: enzyme – trypsin or chymotrypsin; missed cleavages – up to 1 for trypsin and up to 5 for chymotrypsin; fixed modifications – carbamidomethyl (C); variable modifications – oxidation (M), phosphorylation (ST); peptide mass tolerance – 10 ppm; fragment mass tolerance – 20 mmu. The false discovery rate (FDR) threshold for peptide-spectrum matches was set to 1%. phosphoRS 3.0 algorithm [67] was used to assess probability of p-site localizations.

### Pull-down assays

To dissect protein-protein interactions, 20 μg of GST-tagged baits were mixed with equimolar amount of Hrr25 variants in pull-down buffer containing 20 mM Tris, pH 7.5, 150 mM NaCl, 0.5% v/v Tween 20, 2 mM DTT, and 2 mM MgCl_2_. Input controls were taken after mixing of the proteins and prior to addition of GST Sepharose 4B (Sigma). Protein mixtures were incubated at 4°C for 1 h with ∼30 μl beads slurry pre-equilibrated in pull-down buffer. Next, samples were spun at 500 × *g* for 1 min, supernatants removed, and beads washed with pull-down buffer. After three washes, residual proteinswere denatured in Laemmli sample buffer for 5 min at 95°C.

### Thermal shift assays

To analyse denaturation profiles of Hrr25 protein before and after autophosphorylation and the influence of nucleotides on protein stability, 7 μg of full length Hrr25 protein were placed in 20 mM Tris, pH 7.5, 150 mM NaCl, 2 mM DTT, 2 mM MgCl_2_ buffer, 1 mM of corresponding nucleotide and 1× SYPRO Orange (Sigma–Aldrich) hydrophobic fluorescent dye. Samples were slowly heated up from 4°C to 98°C at a rate of 0.3°C/min. Denaturation-related increase in fluorescence was monitored after each heating step using Bio-Rad CFX96 thermocycler. Highlighted inflection points were determined from the first derivative. Data were acquired in three independent experiments with at least two technical replicates each.

### Yeast protein extraction, co-immunoprecipitation and western blot analysis

Yeast cells harvested at mid-log phase (OD_600_= 1) were subjected to total protein extraction through mechanical breakage similar as described in [54] but in 1× IPP100 buffer [10 mM Tris–HCl, pH 8, 100 mM NaCl, 0.1% (v/v) NP-40, 1 mM DTT], supplemented with protease and phosphatase inhibitors (Roche, Germany). Protein concentrations were determined via Bradford assay [68]. Immunoprecipitation (IP) of c-Myc-tagged Elp1 was carried out using anti-c-Myc antibody (9E10, Santa Cruz Biotechnology, USA) coupled to magnetic beads (Thermo Fisher Scientific, USA) according to manufacturer’s instruction. 6.5 mg protein was incubated with 3 μg antibody-coupled beads for 16 h with end-over rotation at 4°C. Antibody bound fractions were collected using a magnetic rack and washed thrice with 1× IPP100 buffer. Elution of proteins was carried out through incubation for 15 min at 50°C in elution buffer [50 mM Tris–HCl, pH 8, 0.2% sodium dodecyl sulphate (SDS), 0.1% Tween^®^ 20], followed by boiling in 1× Laemmli buffer for 5 min at 99°C [69]. Protein samples were separated by SDS–PAGE and analysed by western blotting using anti-c-Myc (9E10, Thermo Fisher Scientific, USA), anti-Hrr25 (EurogenTec, Belgium), anti-HA (Dianova, Germany), and anti-Cdc19 (kindly provided by Dr J. Thorner, University of California, Berkley, CA, USA) antibodies. Eventually, SDS gels were subjected to Coomassie staining for 1 h in protein gel staining solution. Analysis of protein phosphorylation status involved Phos-tag™ (100 μM; NARD, Japan) supplemented to conventional SDS–PAGE [8% acrylamide (acrylamide/bisacrylamide 29.2:0.8)] and 200 μM MnCl_2_, and followed by western blot (WB) analysis according to [70].

### Generation and usage of anti-Hrr25 antibody

The anti-Hrr25 antibody was custom-made by Eurogen Tec. For immunization of rabbits, recombinant Hrr25 1–394 was used as antigen. From the final bleed, the antibody was extracted using Magne^®^ Protein A Beads (Promega, Walldorf), according to manufacturer’s instruction. Therefore, 100 μl serum was incubated with 20 μl prior washed beads for 1 h at 4°C under constant rotation (12 rpm). Subsequently, the beads were collected by centrifugation (500 × *g*, 1 min) and collected using a magnetic rack (Thermo Fisher). Beads were washed thrice with 500 μl 0.1 M Tris, pH 8. After the final washing step, 50 μl 0.1 M glycine pH 2.8 was added and beads incubated for 15 min. Beads were collected, the supernatant was transferred into a new tube and the elution step was repeated. The supernatant was pooled and the pH was adjusted with 20 μl 2 M Tris, pH 8. The antibody solution was diluted 1:2 with 100% (v/v) glycerol, IgG concentration was estimated (237.75 μg/ml), and the solution stored at −20°C. WBs used a 1:3000 dilution of the anti-Hrr25 antibody.

### Protein visualization and bioinformatics

Protein structures were visualized using PyMOL software [71]. Multiple sequence alignments were carried out in the JalView software [72]. ConSurf server [73] was used to visualize evolutionary conservation of protein surface, while APBS electrostatics aided the estimation of surface charge distribution [74]. Generation of phylogenetic trees were performed using AlignX of Vector NTI Advance^®^ 11.0 (Thermo Fisher, 2008).

## Results

### Hrr25 kinase domain mutations cause pleiotropic traits and cell growth defects

**Figure 1.**
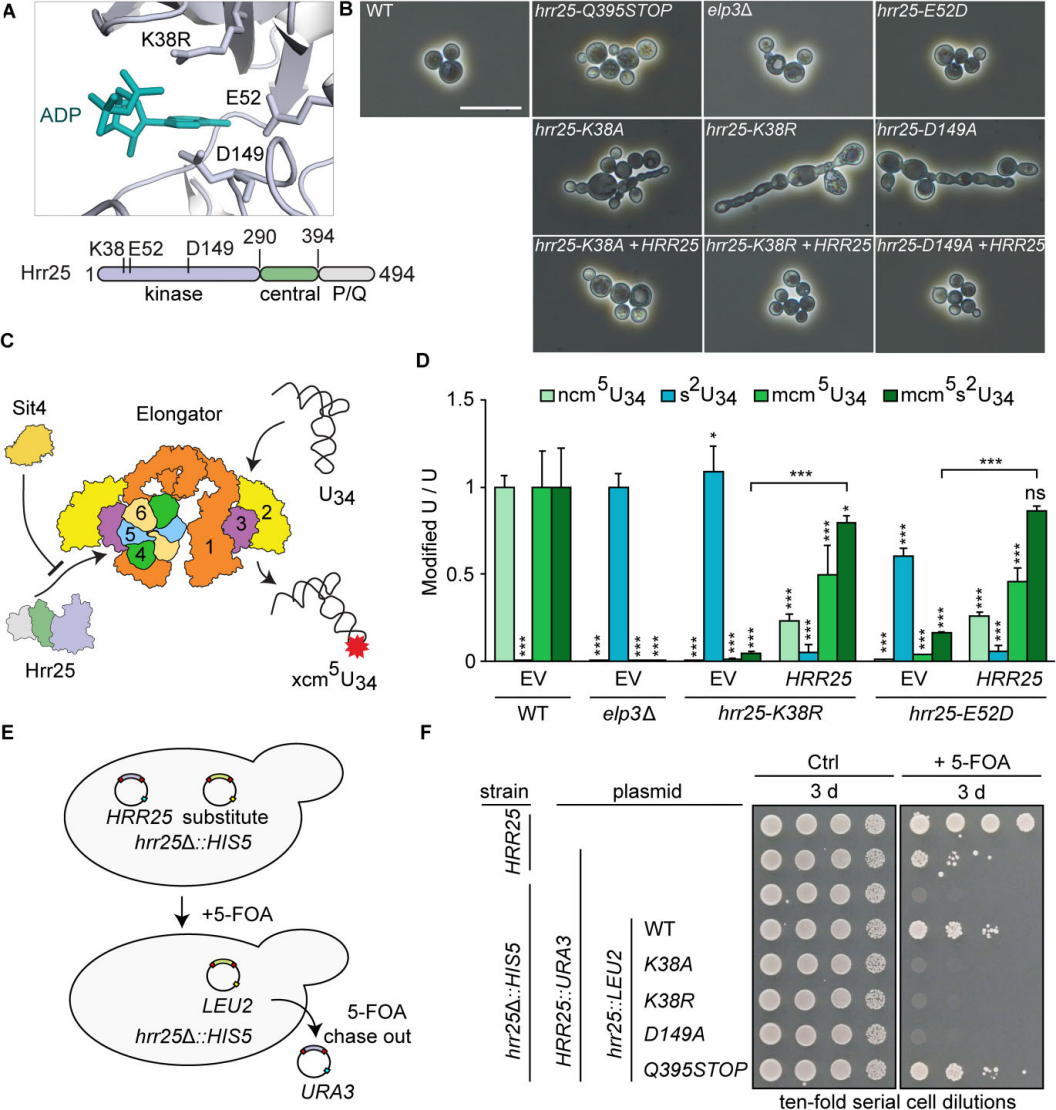
Hrr25 is crucial for yeast cell morphogenesis and viability. (**A**) Scheme of Hrr25 domain architecture with emphasis on the ADP bound catalytical triad (PDB 4xhg). (**B**) Phase contrast microscopy images and morphology of exponentially growing yeast strains with indicated genetic backgrounds (bar: 20 μm). (**C**) The Elongator complex, which catalyses U_34_ modification of tRNA (xcm^5^U), is regulated by Hrr25 kinase and Sit4 phosphatase. (**D**) LC-MS/MS profiles of U_34_ modification states from the indicated strain backgrounds. Modified nucleoside signal was normalized using the total uridine (**U**) content. ncm^5^U, mcm^5^U, and mcm^5^s^2^U signals were normalized against WT, the s^2^U signal against *elp3*Δ. The statistical significance was tested with a two-tailed *t*-test (***P <.001, **P <.01, *P <.05, ns > 0.05). EV, empty vector; ns, not significant. (**E**) Gene shuffle scheme based on 5-FOA plasmid chase-out assay. A lethal *hrr25*Δ allele is rescued by *HRR25* on a *URA3* plasmid that can be selected against by 5-FOA if another plasmid (*HRR25*::*LEU2*) is present as substitute. (**F**) Gene shuffles show Hrr25 catalytic activity is essential for *hrr25*Δ rescue and cell viability. Ten-fold serial cell dilutions of indicated strains were replica spotted on control (Ctrl) medium without or with 5-FOA and cultivated for 3 days at 30°C.

The yeast CK1 isozyme Hrr25 is composed of an N-terminal kinase domain (aa1–290), a central segment (aa291–394), and a C-terminal region (aa395–494) rich in proline and glutamine (P/Q) residues (Fig. 1A). To study Hrr25 function *in vivo*, we compared a genomic P/Q truncation (*Q395STOP*) with substitutions in the kinase domain (*K38A/R*, *E52D*, *D149A*), which were previously shown to impair CK1 activity *in vivo* and *in vitro* (Fig. 1A) [10, 14–16, 46]. Parental *HRR25* WT, *E52D*, or P/Q truncation (*Q395STOP*) strains exhibit proper morphology (Fig. 1B). However, catalytic inactivation of *hrr25* by *K38A*/*R* and *D149A* mutations caused morphological defects, including atypical budding and clusters of elongated cells, which are fully suppressed by ectopic expression of *HRR25* (Fig. 1B).

To check if these phenotypic differences originated directly from Hrr25 catalytic inactivation or downstream CK1 substrates such as the Elongator complex [35] we assessed the morphology of an *elp3*Δ strain, which showed no abnormalities (Fig. 1B). Aberrancies similar to the kinase-dead mutants (*K38A*/*R*; *D149A*) were also observed under conditions of chemical CK1 inhibition using an *hrr25* gate-keeper mutant (*I82G*) [75] with an enlarged binding pocket for bulky, nonhydrolysable ATP analogues (i.e. 1-NM-PP1; 3-MB-PP1) [76] (Supplementary Fig. S1A).

Hrr25 kinase activity is necessary for the modification of tRNA anticodons at wobble uridines (U_34_) by the Elongator complex [35, 46, 49] (Fig. 1C). Consistently, mass spectrometry (MS) showed that CK1 mutants (*K38R*, *E52D*) drastically decreased Elongator-dependent U_34_ modifications *in vivo* (i.e. 5-carbamoyl-methyluridine, ncm^5^U_34_; 5-methoxycarbonyl-methyluridine, mcm^5^U_34_; 5-methoxycarbonyl-methyl-2-thiouridine, mcm^5^s^2^U_34_) and led to an accumulation of a thiolation (s^2^U_34_) usually not found in yeast tRNAs from WT cells (Fig. 1D) [35, 49, 77, 78].

These effects are similar to an Elongator mutant (*elp3*Δ), which is unable to properly modify U_34_ (Fig. 1D). In further support of a direct link between CK1 and Elongator activity, LC-MS/MS shows that the U_34_ modification defects of *hrr25* mutants were rescued by the *HRR25* WT gene (Fig. 1D). Moreover, all *hrr25* mutants including *Q395STOP, K38A*, and *D149A*, survived growth inhibition *in vivo* by zymocin, a tRNase toxin (Supplementary Fig. S1B), which cleaves Elongator modified tRNAs [64, 79, 80]. Of note, isolated tRNAs from all the *hrr25* mutant strains (*Q395STOP, K38A/R*, *E52D*, *D149A*) were also protected against the tRNase cleavage *in vitro* (Supplementary Fig. S1C) indicating tRNA modification defects typical of Elongator-minus cells (*elp3*). These observations are consistent with another read-out showing that *nonsense* suppressor tRNA^Tyr^ (*SUP4*), which requires Elongator-dependent U_34_ modification for read-through of *ade2-1*^ochre^ and adenine prototrophy [49, 81], fully depends on cellular Hrr25 kinase activity (Supplementary Fig. S1D). The observation that normal morphology is retained in *elp3* and some *hrr25* mutants defective in Elongator activity (*Q395STOP, E52D*) reveals that cellular abnormalities in *hrr25 K38A/R* and *D149A* are linked to kinase defects rather than to the inactivation of the Elongator complex.

Given that loss of Hrr25 (*hrr25*Δ) is lethal to haploid yeast strains, we next compared the impact of the kinase-dead mutations on cell viability. Using a *HRR25* gene shuffle approach for *hrr25*Δ complementation analysis (Fig. 1E), *LEU2* plasmids encoding a P/Q truncation (*Q395STOP*) or WT kinase (*HRR25*) were found to support *hrr25*Δ viability upon 5-FOA chase-out of the balancer plasmid (*HRR25*::*URA3*), while those with kinase-dead (*K38A*/*R*, *D149A*) alleles failed to do so (Fig. 1F and Supplementary Fig. S1E). To correlate *hrr25* complementation with kinase expression *in vivo*, anti-Hrr25 WBs showed that CK1 mutant levels compare to Hrr25 WT expression (Supplementary Fig. S1F). In summary, our data indicate that the integrity of the kinase domain, which is necessary for phosphorylation, promotes Elongator dependent tRNA modifications and is important for cell morphogenesis and viability.

### Mapping Hrr25 p-sites on Atg19, Atg34, and Elp123

**Figure 2.**
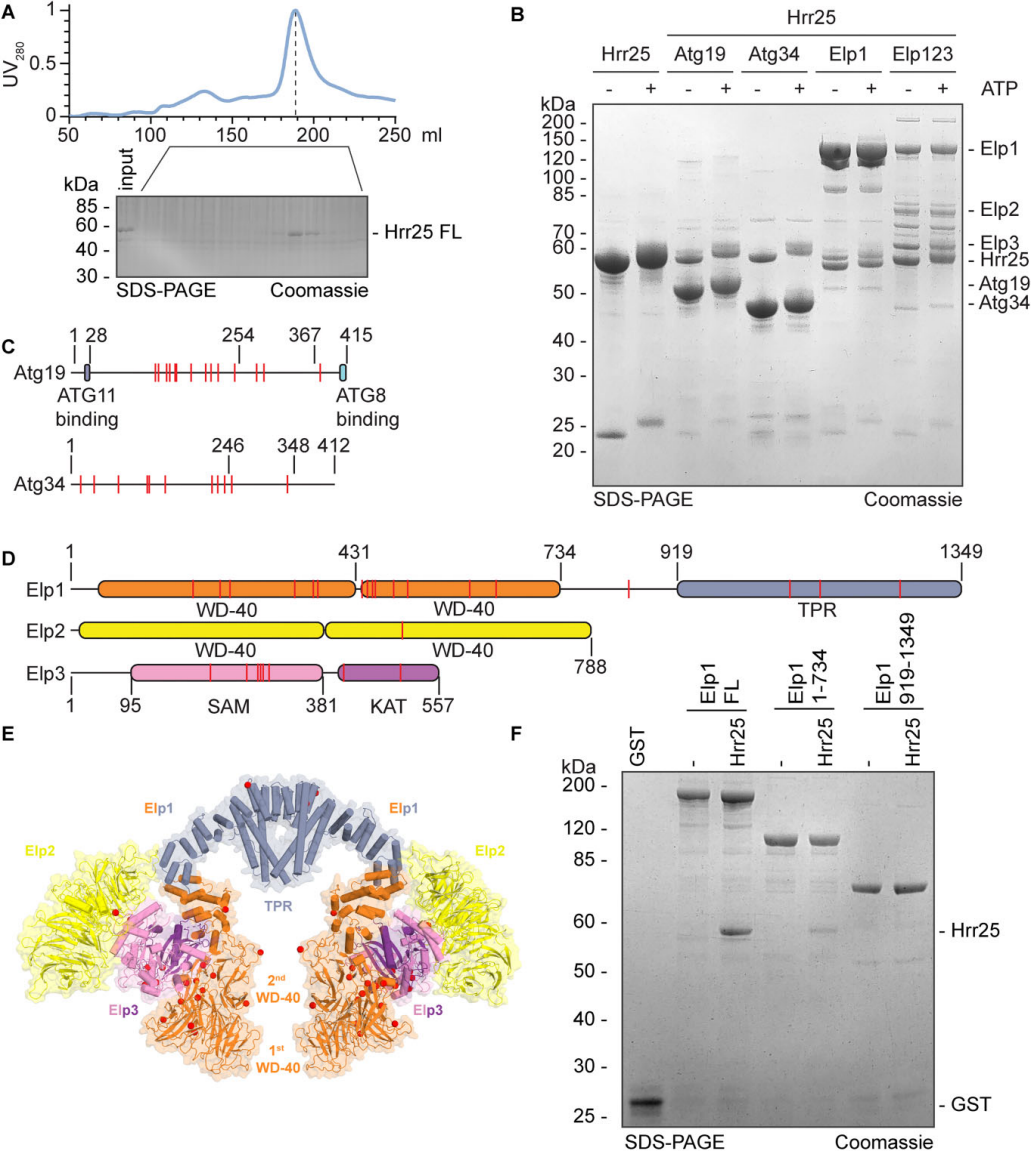
Mapping of p-sites on Atg19, Atg34, and Elp123. (**A**) Representative size exclusion chromatography profile and SDS–PAG E gel of FL Hrr25 purified from Hi5 cells. Ultraviolet signal is scaled to 1. (**B**) Hrr25 FL is capable of *in vitro* phosphorylation. All samples were incubated for 1 h at 30°C. Conditions without ATP were used as control. Gel bands were excised from the gel and subjected to MS for identification of p-sites. (**C**) Domain representation of Atg19 and Atg34 with p-sites highlighted as red lines. (**D**) Subunit domain representation of the Elp123 subcomplex. Hrr25-dependent phosphorylations are plotted as red lines. (**E**) Structural overview of p-sites on the Elp123 subcomplex identified in this study. Colouring as in panel (D). (**F**) *In vitro* GST pull-down assay. The N-terminus of Elp1 is the main docking site of Hrr25 on the Elongator complex. GST-Elp1 constructs were used as a bait, free GST was used as a control.

Hrr25 phosphorylates various protein substrates *in vivo*, including Elongator and autophagy receptors (e.g. Atg19, Atg32, Atg34, Atg39) [21, 23, 35, 46]. Next, we purified full-length (FL) yeast Hrr25 from Hi5 insect cells (Fig. 2A) to study CK1 activity *in vitro* using phosphorylation assays for purified Atg19, Atg34 or the Elp123 subcomplex. Attempts to purify other Atg proteins were unsuccessful. We confirmed phosphorylation by electrophoretic band shifts in standard SDS–PAGE and optimized the assay conditions prior to p-site identification by MS (Fig. 2B). The results show that Hrr25 phosphorylates Atg19 *in vitro* on Ser119, Ser124, Ser136, Ser141, Ser149, Thr150, Ser151, Ser173, Thr195, Ser203, Thr214, Thr239, Ser272, Ser283, and Ser368. Atg34 is phosphorylated on Thr7, Thr28, Ser66, Ser111, Ser114, Ser139, Ser212, Ser221, Ser232, Thr243, and Thr330 (Fig. 2C). Many of these p-sites in Atg19 and Atg34 map close to domains that bind Atg11 (Fig. 2C), an adaptor scaffold protein required for autophagy. This is in line with the view that autophagy receptor phosphorylation by Hrr25 enables a direct contact with Atg11 prior to formation of proteolytic autophagosomes [82].

Next, we analysed *in vitro* p-sites in the purified Elp123 subcomplex, which is known to be a substrate for Hrr25 *in vivo* [35]. In case of Elp1, p-sites were mapped to Ser177, Ser218, Thr233, Ser331, Ser359, Ser366, Thr433, Thr441, Thr449, Thr453, Ser481, Ser502, Thr596, Ser636, Thr837, Ser1081, Ser1127, and Thr1248 (Fig. 2D). In Elp2, one p-site was detected at Thr494 and in Elp3, the enzymatic core of Elongator, Ser203, Ser258, Ser275, Thr279, Ser283, Ser292, Thr405, and Ser491 are phosphorylated by Hrr25 *in vitro*. Recently, we determined the structure of yeast Elongator by single particle cryo-electron microscopy (cryo-EM) [61, 83] enabling visualization of the exact location of the p-sites identified in the Elp123 subcomplex (Fig. 2E). A few of them map to the C-terminal tetratricopeptide repeat (TPR) domain in Elp1, responsible for dimerization and previously reported to become phosphorylated by Hrr25 *in vivo* [35, 84, 85]. Nonetheless, the majority of p-sites map to a pocket between the two N-terminal WD40 domains of Elp1 and the catalytically crucial lysine acetyl transferase (KAT) domain of Elp3 (Fig. 2E). Using GST-pull down assays, we confirmed a weak direct interaction between the N-terminal region of Elp1 and Hrr25 (Fig. 2F). In summary, our data provide a comprehensive map of p-sites in different Hrr25 substrates with roles in autophagy (Atg19, Atg34) and tRNA modification (Elp123) that are important for protein homeostasis [82, 86].

### Gene shuffles reveal human CK1ϵ is a functional ortholog of Hrr25 in yeast

**Figure 3.**
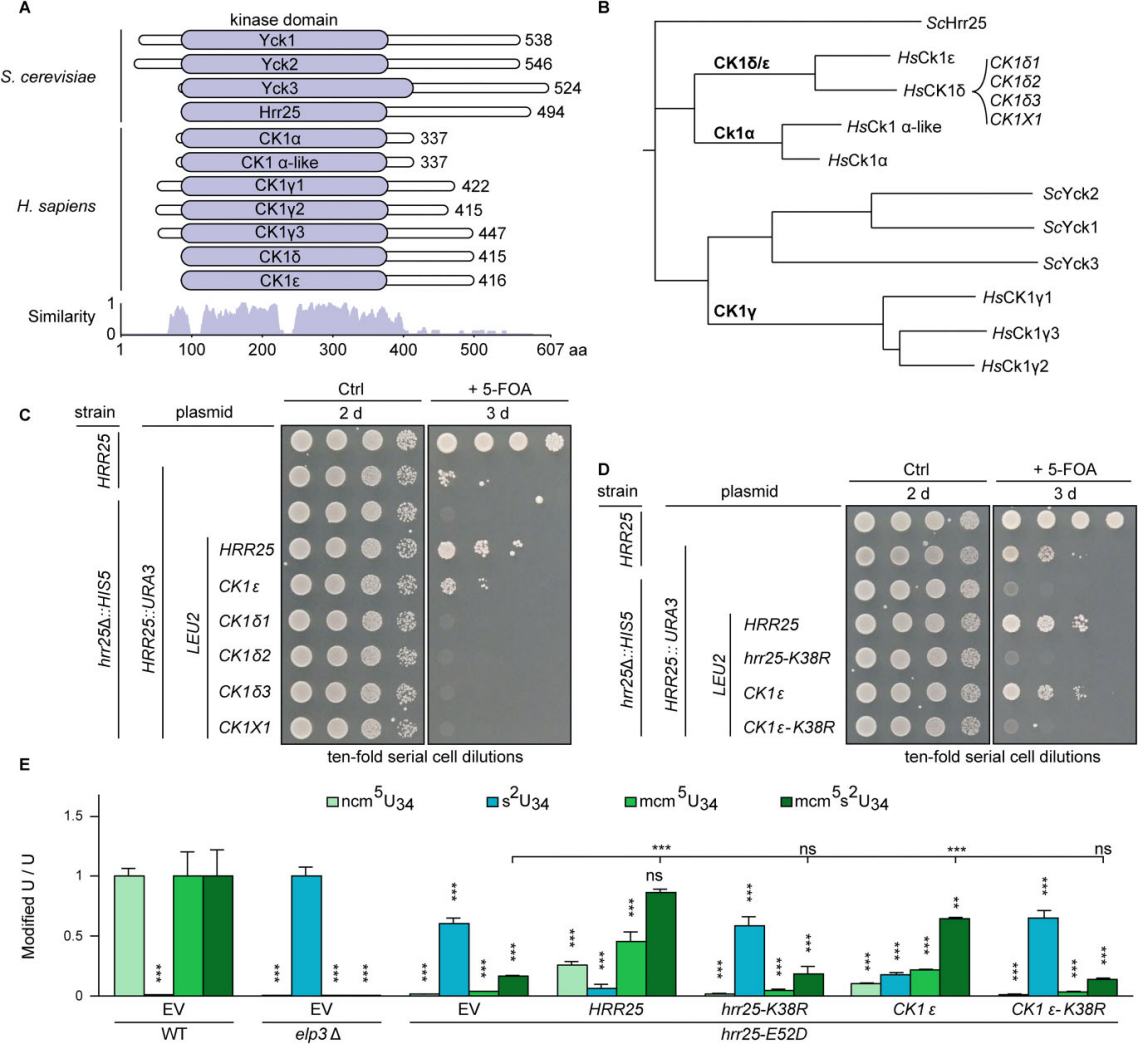
CK1ϵ is the human homolog of Hrr25. (**A**) Multiple sequence alignment of human and yeast CK1. All isozymes share a highly conserved kinase domain (violet) of comparable size and location. (**B**) A phylogenetic tree of human and yeast CK1 modified after [87] and [88]. Branch length is proportional to evolutionary distance. (**C, D**) 5-FOA chase-out assays. Solely, human CK1ϵ complements lack of Hrr25 function and CK1ϵ catalytic inactivation (*K38R*) abolishes *hrr25* mutant complementation *in vivo*. (**E**) LC-MS/MS profiles to demonstrate rescue of U_34_ modification defects in *hrr25* mutant (*E52D*) by CK1ϵ. Signals for modified nucleoside were standardized with total uridine (U) content. ncm^5^U, mcm^5^U, and mcm^5^s^2^U signals were normalized against WT, s^2^U signals against *elp3*Δ. Statistical significance is based on two-tailed *t*-test with *P*-values as in Fig. 1. EV, empty vector; ns, not significant.

CK1 family members share a high degree of amino acid sequence identity within their kinase domains, while other regions are more divergent (Fig. 3A). To identify the most likely Hrr25 ortholog in humans, we performed multiple sequence alignments and calculated a rooted phylogenetic tree (Fig. 3B and Supplementary Fig. S2). Yeast Yck1–Yck3 and human CK1γ1–CK1γ3 sequences form tight species-specific clusters with a relatively short evolutionary distance between them, indicating that they are part of an equivalent isoform group. Yeast Hrr25 exhibits shorter evolutionary distances to human CK1α, CK1δ, and CK1ϵ proteins than to Yck1–Yck3. After calculating pairwise amino acid identity scores from the multiple sequence alignments, we found that Hrr25 shares a higher degree of amino acid identity with CK1δ and CK1ϵ than CK1α (Supplementary Fig. S3A). This comparison reconfirms previous notions [9, 10, 12] that Hrr25 may represent an ortholog of the CK1δ and CK1ϵ families. Consistent with its more distant relationship to Hrr25, CK1α was previously found to fail in complementing yeast *hrr25* deletion [9].

We experimentally addressed this issue by 5-FOA chase-out assays. In detail, we found that after substituting Hrr25 with various human CK1δ and CK1ϵ isozymes, only the CK1ϵ gene copy (and *HRR25* as positive control) allowed the yeast *hrr25*Δ reporter strain to grow without an *HRR25*::*URA3* balancer plasmid (Fig. 3C). None of the genes coding for CK1δ splicing variants promoted cell survival, although they were expressed (Supplementary Fig. S4). To examine whether the observed rescue specifically depends on the kinase activity of CK1ϵ, we used gene shuffles to compare *hrr25*Δ complementation capacity between kinase-dead (*K38R*) isoforms of yeast Hrr25 and human CK1ϵ (Fig. 3D). Upon 5-FOA chase-out, both, the kinase-dead (*K38R*) CK1ϵ isoform as well as its yeast mutant (*K38R*) counterpart, failed to genetically complement and support viability of the *hrr25*Δ mutant (Fig. 3D). Therefore, our data show that CK1ϵ can replace Hrr25 function in yeast cells, instead of the previously anticipated CK1δ [10, 12, 15, 46]. Importantly, its capacity to do so strictly depends on the CK1ϵ kinase domain and its phosphorylation activity.

**Figure 4.**
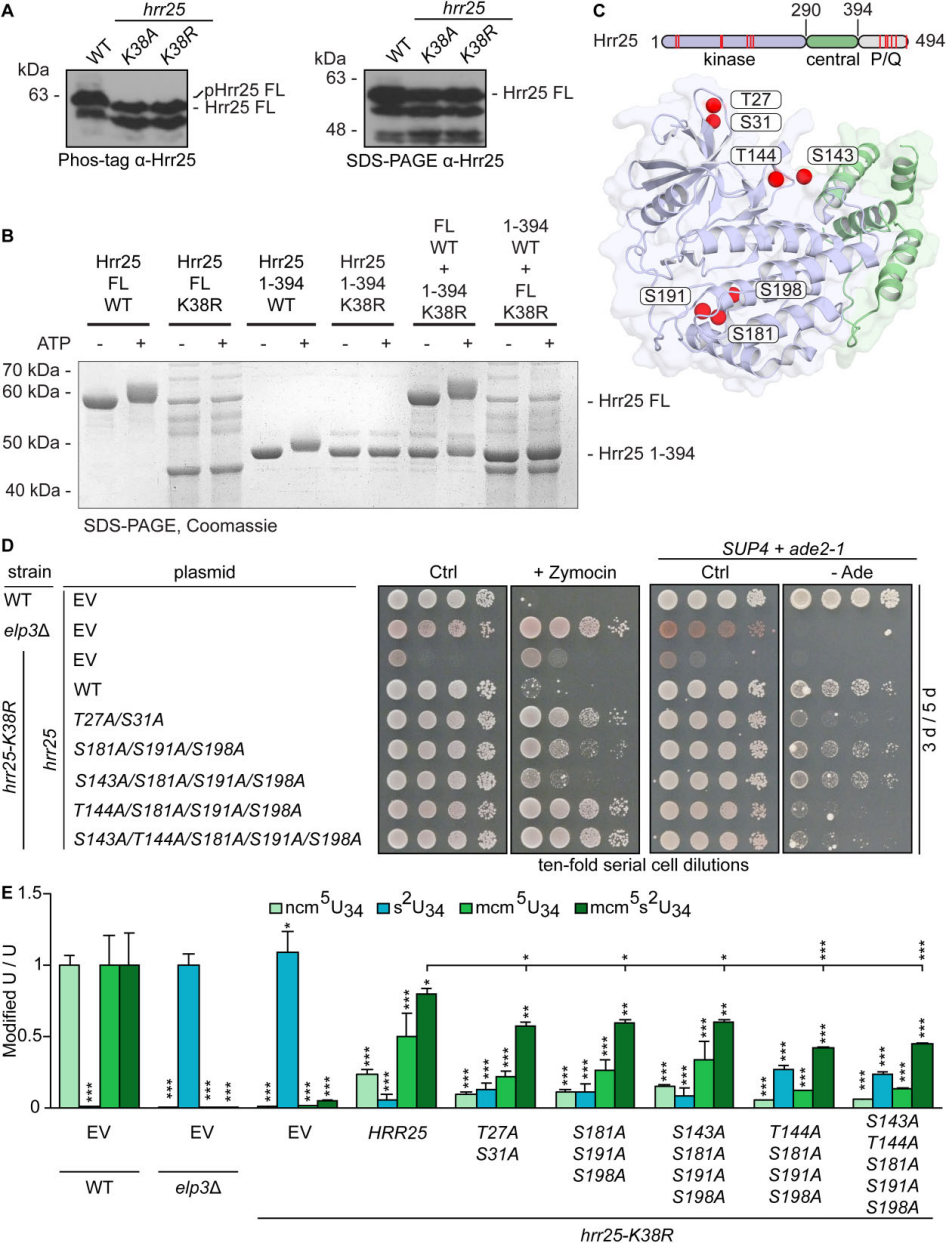
Hrr25 autokinase activity. (**A**) Hrr25 autophosphorylation *in vivo*. WB analysis of yeast lysates obtained from indicated strains and subjected to Phos-tag™ (left panel) or conventional SDS–PAGE (right panel). (**B**) Hrr25 autophosphorylation *in vitro*. Purified FL Hrr25 and Hrr25_1-394_ and their respective *K38R* mutants were incubated with or without ATP for 1 h at 30°C and subjected to SDS–PAGE followed by Coomassie staining. (**C**) Identification of *in vitro* autophosphorylation sites of Hrr25 by MS. Structural overview of detected p-sites (red spheres) plotted on the Hrr25_1-394_ structure (PDB 5cyz). (**D**) p-site mutagenesis shows Hrr25 autophosphorylation mediates zymocin sensitivity and *SUP4* read-through. Serial cell dilutions of the indicated strains were replica spotted on medium lacking (control, Ctrl) zymocin or containing 100% (v/v) (+ Zymocin). For the *SUP4* assay, that monitors *ade2-1^ochre^* read-through, strains were replica spotted on medium containing (Ctrl) or lacking adenine (−Ade). Cultivation was for 3–5 days at 30°C, respectively. (**E**) LC-MS/MS reveals *hrr25* phosphoablative mutations trigger progressive U_34_ tRNA modification defects. For modification measurements and statistical significance analysis, see Figs 1 and 3. EV, empty vector.

We next compared the impact of human CK1 isozymes on the Elongator pathway for tRNA modification, which we showed above is compromised in *hrr25* mutants (Fig. 1D). Based on LC-MS/MS profiles, CK1δ1, CK1δ2, CK1δ3, and CK1 × 1 as well as the kinase-dead (*K38R*) CK1ϵ failed to rescue the tRNA modification defects of the *hrr25* (*E52D*) mutant, whereas catalytically active versions of human CK1ϵ or yeast Hrr25 did (Fig. 3E and Supplementary Fig. S3C). To corroborate cross-species compatibility between CK1ϵ and Hrr25, we also analysed other cellular phenotypes that are associated with Elongator activity, namely temperature sensitivity, zymocin toxicity, and *ochre* read-through by the suppressor tRNA *SUP4*. In all assay formats (Supplementary Fig. S3B and D), CK1ϵ could replace Hrr25 and supported growth processes previously shown to require the highly conserved Elongator pathway for tRNA modification.

To test whether the C-terminal domains of CK1δ and ϵ influence the abilities to complement yeast *hrr25*, we generated truncated forms of both proteins containing the kinase domains alone and tested their ability to complement *hrr25*. While the kinase domain of CK1ϵ weakly complemented *hrr25Δ*, this was not the case for CK1δ (Supplementary Fig. S5A). Hence, the C-terminal domain of CK1ϵ improves *hrr25* complementation, whereas the counterpart of CK1δ is not solely responsible for absence of complementation. Also, we tested whether the kinase domain of Hrr25 alone (1–290) can complement an *hrr25* mutation. Similar to the CK1ϵ kinase domain, a weak complementation was observed (Supplementary Fig. S5A). While these mutants can compensate the growth defect of the *hrr25* deletion, Elongator function cannot be restored by only expressing the kinase domains of Hrr25 and CK1ϵ, as indicated by the zymocin and *SUP4* assay (Supplementary Fig. S5B).

**Figure 5.**
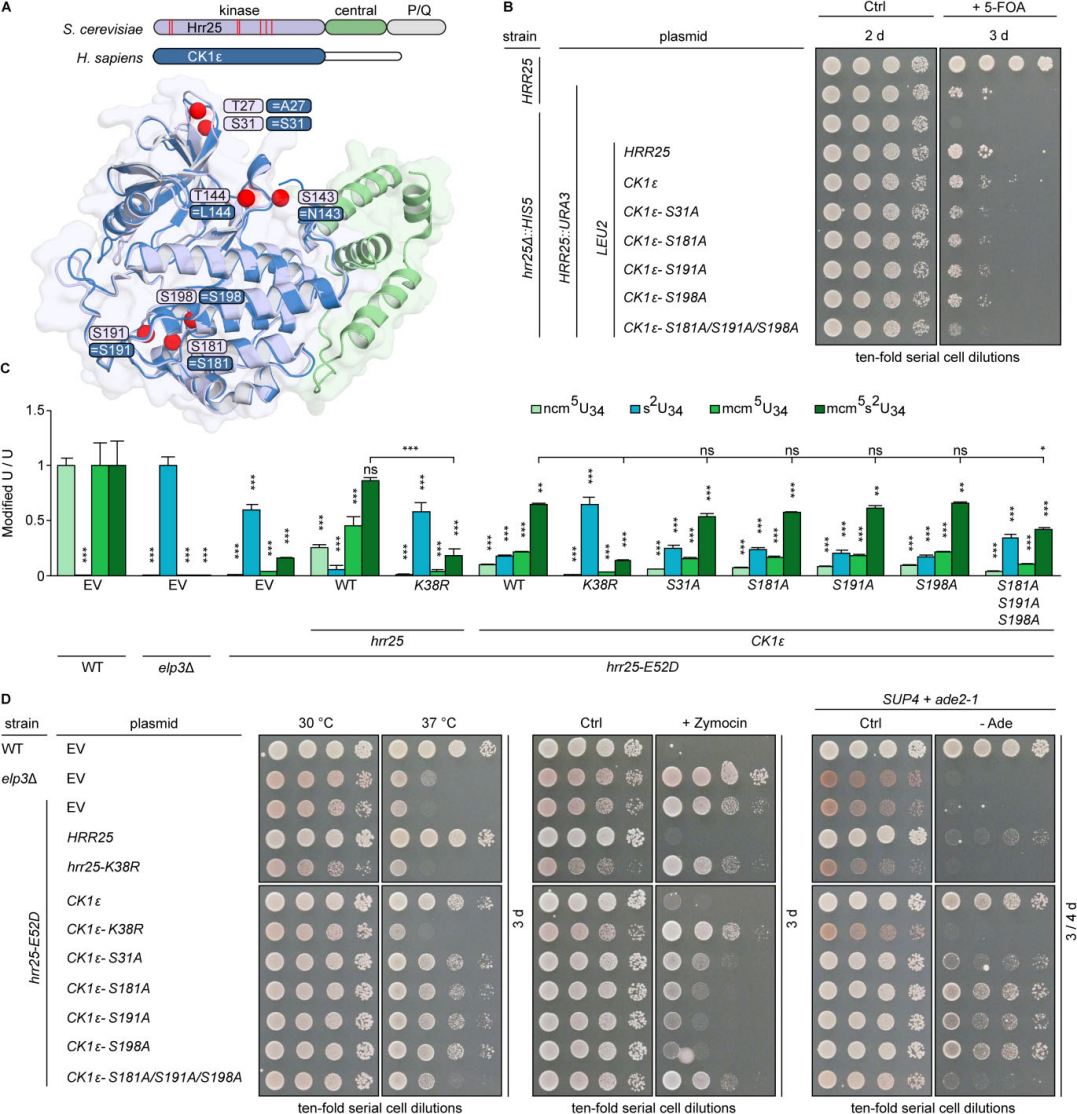
Conservation of Hrr25 p-sites in CK1ϵ. (**A**) Structural alignment of Hrr25 and CK1ϵ identifies conserved autophosphorylation sites. Structures of Hrr25 (PDB 5cyz, 1–394; purple, kinase domain; green, central domain) and CK1ϵ (PDB 4hni, 1–294; kinase domain, blue) were aligned using PyMOL. Positions of conserved p-sites in Hrr25 (purple) and CK1ϵ (blue) are indicated by red spheres. (**B**) Cross-species gene shuffle assay. The capacity of indicated single and multiple CK1ϵ p-site mutations to rescue the inviability of *hrr25*Δ mutant was tested using the 5-FOA chase-out assay as detailed in Figs 1 and 3. (**C**) LC-MS/MS shows lack of U_34_ modification caused by *hrr25* mutant (*E52D*) can be partially substituted by CK1ϵ p-site mutants. For modification measurements, ncm^5^U, mcm^5^U, mcm^5^s^2^U, and s^2^U normalization against WT or *elp3*Δ and statistical significance analysis, see Figs 1 and 3. EV, empty vector; ns, not significant. (**D**) Progressive loss of phenotypic rescue of *hrr25* mutant (*E52D*) by CK1ϵ p-site mutants. Serial dilutions of the indicated strains were grown under conditions to assay rescue of thermosensitivity at 37°C, zymocin resistance and loss of *SUP4* read-through (for details, see Fig. 1 and Supplementary Figs S1 and S3, respectively).

### Hrr25 undergoes functionally relevant autophosphorylation

In addition to substrate phosphorylation, Hrr25 is also capable of autophosphorylation, a feature CK1 isozymes share with many other protein kinases [11, 32, 89, 90, 91]. To study Hrr25 autophosphorylation, we applied total yeast extracts to SDS–PAGE containing Phos-tag [70] and performed WBs using anti-Hrr25 antibodies. A clearly visible electrophoretic mo-

bility shift between Hrr25 from WT (*HRR25*) and kinase-dead (*K38A/R*) cells suggests CK1 autophosphorylation *in vivo* (Fig. 4A). Using purified FL Hrr25 and the P/Q truncation (Hrr25 1–394) (Supplementary Fig. S6A), we reconstituted the autophosphorylation *in vitro* and found that it strictly depends on the active site of the CK1 isozyme, since purified kinase-dead (*K38R*) versions (FL or 1–394) lacked autophosphorylation capacity *in vitro* (Fig. 4B). We confirmed that phosphorylation underlies the observed electrophoretic mobility shifts by treatment of the samples with alkaline phosphatase that reverses the respective shifts (Supplementary Fig. S6B).

**Figure 6.**
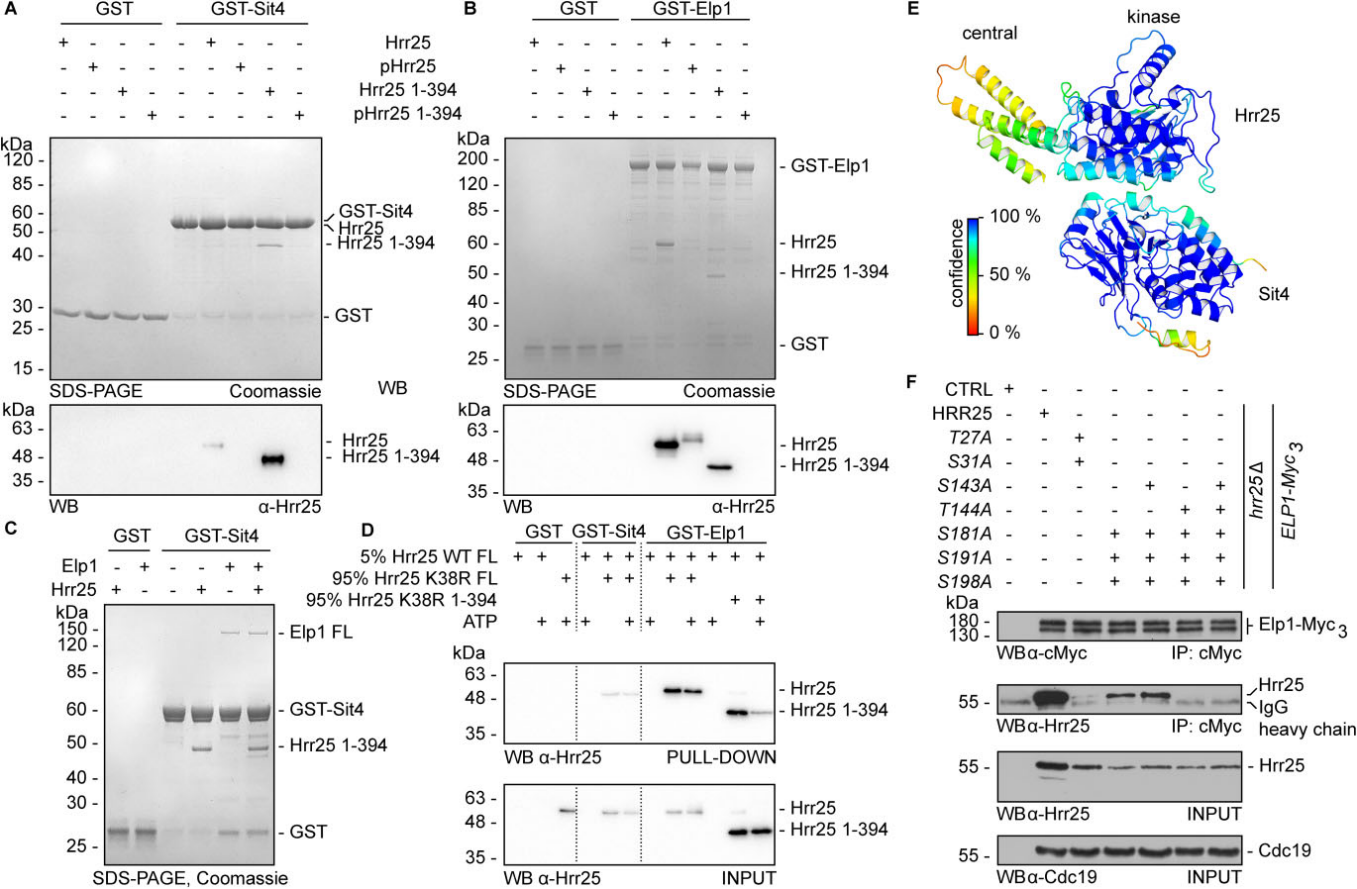
Hrr25 autophosphorylation defines kinase interactions with Elp1 and Sit4. (**A, B**) GST pull-down assay addressing Hrr25–Sit4 (**A**) and Hrr25–Elp1 (**B**) interactions. GST-Sit4 and GST-Elp1 were used as a bait, free GST served as a negative control. Interaction was visualized with SDS–PAGE (top) and WB with Hrr25-specific antibodies (bottom). (**C**) GST pull-down assay showing interaction between Sit4 and Elp1 is not mutually exclusive with Hrr25 binding. (**D**) *trans*-autophosphorylation of Hrr25 does not exert strong influence on Hrr25–Sit4 interaction, but significantly impairs Elp1 interaction. WB analysis of GST pull-down (top membrane) and input (bottom membrane). The 5% of catalytically active Hrr25 WT provided *trans*-autophosphorylation to 95% of Hrr25 K38R incapable of autophosphorylation. Hence, the majority of readouts corresponds to the effect of autophosphorylation in *trans*. (**E**) AlphaFold model of Hrr25–Sit4 complex coloured according to confidence. (**F**) Autophosphorylation positively regulates kinase interaction with Elp1. Proteins were isolated from indicated strains and subjected to anti-c-Myc IP followed by WB analysis of either input control or coprecipitated proteins. To assure nonspecific binding to the beads, a control without protein was included Immunodetection of Myc-tagged Elp1 and Hrr25 employed anti-c-Myc or anti-Hrr25 antibodies. Cdc19 detection served as protein loading control.

To check whether autophosphorylation occurs in *cis* or *trans* [92, 93], we mixed active, FL Hrr25 with a truncated kinase-dead (*K38R*) version lacking the P/Q domain, Hrr25_1–394_, and noticed that both versions show phosphorylation-dependent retardation (Fig. 4B). When the FL kinase-dead version was mixed with truncated Hrr25 containing the kinase active site, no autophosphorylation was detected (Fig. 4B). Although a part of the phosphorylation events targeting the kinase-dead P/Q truncation occurs in *trans*, autophosphorylation of FL active Hrr25 kinase in *cis* cannot be excluded. Irrespective of the precise mode, the presence of ATP increased Hrr25 thermostability, and kinase re-purified post-autophosphorylation *in vitro* showed a similar increase in melting temperature (Supplementary Fig. S6C). This strongly suggests autophosphorylation confers stability to the yeast CK1 isozyme.

Next, we used MS to map Hrr25 p-sites after incubation of purified active kinase with ATP. In addition, we analysed p-sites of a kinase-dead (K38R) version to detect phosphorylation events by other kinases from the expression host prior to purification of recombinant Hrr25 material. Thereby, we identified autophosphorylation sites at areas of low electrostatic potential in the kinase domain (Thr27, Ser31, Ser143, Thr144, Ser181, Ser191, Ser198) (Fig. 4C and Supplementary Fig. S6D–F) and within the P/Q rich C-terminus of Hrr25 (Ser438, Thr449, Thr453, Ser461, Ser470; Ser491) (Fig. 4C). To dissect the influence of the P/Q rich C-terminus of Hrr25, we performed a similar MS-based approach to detect the autophosphorylation pattern of Hrr25 1–394. In addition to sites detected in full length Hrr25, we observed phosphorylations at positions Ser41, Ser44, Ser60, Ser153, Thr161, Ser236, Thr239, Ser242, Tyr266, Ser317, Thr326, Ser330, Thr346, Ser376 in the truncated variant. None of these sites were autophosphorylated in full length Hrr25 kinase, suggesting that the P/Q rich C-terminus inhibits autophosphorylation and defines specificity of autophosphorylation by the kinase domain (Supplementary Fig. S6A). We decided to focus our attention on autophosphorylation events taking place within the kinase domain, since the C-termini of Hrr25 and CK1ϵ are not conserved (Fig. 3A and Supplementary Fig. S2A). Ser181, Ser191, and Ser198 are buried in the Hrr25 core and four other sites (Thr27, Ser31, Ser143, Thr144) are exposed at surface areas of low electrostatic potential (Supplementary Fig. S7). For several of these sites, phosphoablative exchanges were introduced in Hrr25_1_Hrr25_–394_, recombinant proteins purified and subjected to autophosphorylation assays along with active site mutants *K38R* and *K38A* (Supplementary Fig. S7). Noteworthy, almost all autophosphorylation mutants undergo autophosphorylation with the exception of *K38R/A* (catalytic inactivation) and the *S181A/S191A/S198A* triple mutant.

**Figure 7.**
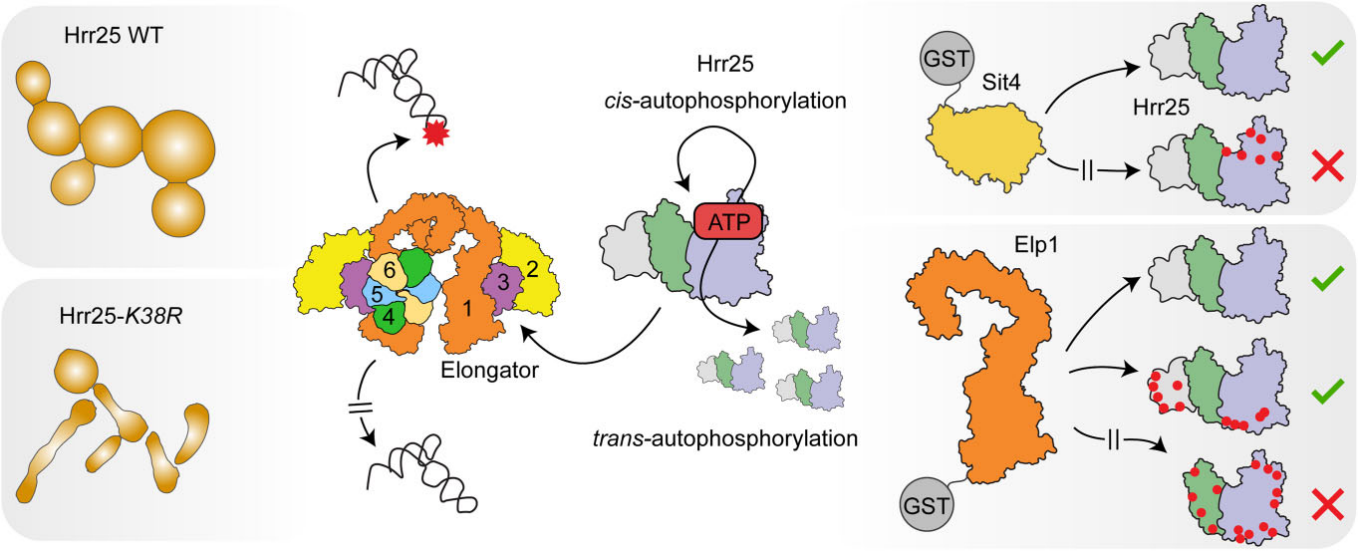
Differential roles of CK1 Hrr25 *cis*- and *trans*-autophosphorylation for the Elongator pathway in yeast.*cis*-autophosphorylation releases the CK1 from a Hrr25–Sit4 complex characterized in this study. Autophosphorylation of Hrr25 in *trans* decreases Hrr25 capacity to bind Elp1, the main scaffolding subunit of the Elongator complex. Upon binding, Hrr25 provides multiple phosphorylation of the Elp123 subcomplex, enabling a faithful tRNA modification process. In yeast, lack of Hrr25 kinase activity (*K38R*) results in pleiotropic phenotypes including major perturbations in cell morphology.

To understand the functional relevance of the detected sites *in vivo*, we substituted the identified p-sites by alanine and introduced plasmid-based alleles into *hrr25* mutant strains. Using the 5-FOA chase-out assays, all substitutions were found to support *hrr25*Δ viability (Supplementary Fig. S8A). Thus, kinase activity in all substitution mutants is sufficient to sustain cell viability in standard growth conditions upon ablation of Hrr25 p-sites. However, under stress, i.e. at 37°C or in presence of cell wall indicator drug CFW, *hrr25-K38R* cell growth was compromised by quadruple/quintuple (*S143A/T144A/S181A/S191A/S198A*) mutations (Supplementary Fig. S8B and C). Thus, the data show that *in vivo*, the activity of Hrr25 depends on upon multiple, redundant p-sites. Consistent with this model, LC-MS/MS tRNA modification profiles from the phosphoablative mutants show that decreased levels of Elongator dependent U_34_ modifications (ncm^5^U_34_; mcm^5^U_34_; mcm^5^s^2^U_34_) and accumulation of the aberrant thiolation (s^2^U_34_) are most prominent in cells carrying the quadruple/quintuple substitutions (Fig. 4E). A negative effect on tRNA wobble uridine modification in the quadruple/quintuple substitution mutant was also supported by increased zymocin resistance and decreased *SUP4* function (Fig. 4D). Since the quadruple/quintuple Hrr25 mutants both involve the *T144A* exchange, we individually tested effects of a single *T144A* mutation on zymocin and *SUP4* phenotypes (Supplementary Fig. S9). As these phenotypes were unaffected by *T144A* alone and only moderately affected by *S181A/S191A/S198A*, autophosphorylation at these sites may be functionally redundant in terms of supporting Elongator activity. Considering that autophosphorylation deficient Hrr25 strains phenocopy *elp3*Δ cells *in vivo* (Fig. 4D), we conclude that a redundant set of autophosphorylation sites in the kinase domain is needed for Elongator dependent tRNA modifications. Thus, Elongator activity *in vivo* is under autophosphorylation control of the yeast CK1 isozyme Hrr25.

### Hrr25 autophosphorylation sites are conserved in human ortholog CK1ϵ

With Hrr25 and CK1ϵ being orthologs capable of cross-specific complementation, we asked whether autophosphorylation is also conserved between yeast and human kinase. Among the seven Hrr25 p-sites identified above (Fig. 4C), four residues (Ser31, Ser181, Ser191, Ser198) are conserved in CK1ϵ (Fig. 5A). To analyse their potential for phosphorylation and relevance *in vivo*, we generated *LEU2* plasmids with single (*S31A*; *S181A*; *S191A*; *S198A*) and triple (*S181A/S191A/S198A*) phosphoablative substitutions for complementation analysis in yeast (Fig. 5B). WT CK1ϵ and each single substitution mutant complemented *hrr25*Δ viability, while triple (*S181A/S191A/S198A*) phosphoablative substitutions in the CK1ϵ mutant hardly did. This finding is well in support of CK1ϵ autophosphorylation at three sites conserved in Hrr25. Moreover, it strongly suggests that ablation of these p-sites in CK1ϵ by genetic means interferes with Hrr25 function and signalling in yeast.

LC-MS/MS profiles show that among the tested strains, the CK1ϵ triple (*S181A/S191A/S198A*) mutant has lowest capability to restore ncm^5^U_34_, mcm^5^U_34_ and mcm^5^s^2^U_34_ modifications and highest s^2^U_34_ levels diagnostic for Elongator dysfunction (Fig. 5C). Although modification levels are not as severely affected as with the kinase-dead (*K38R*) variant of CK1ϵ, they imply that proper complementation capability by CK1ϵ requires conserved p-sites for autophosphorylation. Furthermore, phenotypic assays with the *hrr25-E52D* reporter show that growth defects can hardly or not all be rescued by either triple (*S181A/S191A/S198A*) phosphoablative or kinase-dead CK1ϵ (*K38R*) mutants (Fig. 5D). Similarly, other traits of the *hrr25-E52D* reporter mutant, which the triple (*S181A/S191A/S198A*) mutant failed to complement, include thermosensitivity at 37°C, protection against zymocin tRNase and abolishment of tRNA suppression by *SUP4* (Fig. 5D). In summary, the dependency on autophosphorylation is conserved between CK1 orthologs and required for CK1ϵ to promote Hrr25 signalling and Elongator phosphorylation for the tRNA modification pathway.

### Hrr25 autophosphorylation regulates CK1 interaction with Elongator and Sit4

Although we demonstrated the importance of Hrr25 activity and autophosphorylation state for Elongator function *in vivo*, the molecular events behind this phenomenon remain elusive. As previously shown [46], the phosphorylation state and activity of the Elongator complex are regulated by antagonistic influences of Hrr25 kinase and Sit4 phosphatase under physiological conditions. To shed light on this regulatory circuit we decided to investigate whether Hrr25 kinase or autophosphorylation activity regulates the dynamic interaction of Hrr25 with Elongator subunit Elp1 or the phosphatase Sit4.

In detail, we examined the interaction between Sit4 and Hrr25 *in vitro*, by checking the ability of purified GST-tagged Sit4 to bind nonphosphorylated and autophosphorylated forms of FL kinase or truncation variant Hrr25_1-394_ lacking the P/Q-rich region (Fig. 4B). We detected a direct protein-protein interaction between Sit4 and nonphosphorylated Hrr25 and observed that formation of the complex is inhibited by Hrr25 autophosphorylation (Fig. 6A and Supplementary Fig. S10A). Based on these pull-down experiments, FL Hrr25 may bind Sit4 less efficiently than Hrr25_1–394_ (Fig. 6A) which may point to an inhibitory role of the P/Q rich region. Contrary to the interaction with Sit4, FL Hrr25 binds Elp1 regardless of autophosphorylation (Fig. 6B and Supplementary Fig. S10B). Autophosphorylated Hrr25_1–394_ fails to bind to FL GST-Elp1, but interacts with a truncated form of GST-Elp1 1–734 (Supplementary Fig. S10D). Given that Hrr25 individually interacts with Sit4 and Elp1, we checked whether these interactions are mutually exclusive or can happen simultaneously. We did not observe exclusive binding of either Hrr25 or Elp1, but due to the lowered stoichiometry the simultaneous binding between Sit4, Hrr25, and Elp1 cannot ultimately be confirmed or excluded (Fig. 6C and Supplementary Fig. S10C). Future studies involving purified Sit4-Sap185/190 holo-phosphatase complex may be needed to address this issue.

To test for differences between autophosphorylation in *cis* or in *trans*, we generated a predominantly *trans*-autophosphorylated sample of Hrr25 and probed its interaction capacity with GST-Sit4 and GST-Elp1 (Fig. 6D). Strikingly, we found that *trans*-autophosphorylation no longer abolishes Hrr25 interaction with Sit4 (Fig. 6A and D) and only *cis*-autophosphorylation is able to inhibit kinase association with Sit4. Consistent with previous observations, *trans*-autophosphorylation of Hrr25 did not disrupt interaction with Elp1 (Fig. 6B and D). We used AlphaFold-Multimer v2 to model the potential Hrr25–Sit4 complex (Fig. 6E). In line with our binding studies, Hrr25 and Sit4 are predicted to interact in the vicinity of the Hrr25 active site, where *cis*-autophosphorylation sites are plausible. As the obtained model exhibits relatively low confidence scores at the interface, future structural studies are needed to validate it.

Although Hrr25 might exhibit multiple phosphorylation states, including a nonphosphorylated form together with *cis* and *trans* autophosphorylation or combinations thereof, we checked whether the lack of phosphorylation at particular p-sites could influence Elp1 binding *in vivo* (Fig. 6F). We resorted to IP of Elp1-myc from lysates of

yeast strains expressing various phosphoablative substitutions in Hrr25 (see Fig. 4D) and found that the p-site mutants showed reduced ability to coprecipitate with Elp1 (Fig. 6F). In particular, the double (*T27A/S31A*) and quadruple/quintuple (*S143A/T144A/S181A/S191A/S198A*) substitutions (Fig. 4D) failed to be precipitated with Myc-tagged Elp1 (Fig. 6F). Thus, p-sites located at the Hrr25 surface (Thr27, Ser31, Ser143, Thr144) appear to be crucial for Hrr25 interaction with Elp1, whereas others (Ser181, Ser191, Ser198) are dispensable for kinase association with Elongator.

## Discussion

Using comprehensive molecular analyses *in vivo* and *in vitro*, we show that the kinase domain of Hrr25 is crucial for proper cell morphogenesis and viability. In contrast to any of the three nonessential members of the CK1 gene family in yeast (*YCK1*, *YCK2*, *YCK3*), the *HRR25* locus is indispensable for cell survival in most standard yeast strains. This vital function depends on the catalytic triad (*K38, E52, D149*) and the phosphorylation activity associated with Hrr25. In addition to the *K38R/A* and *E52D* mutations for full and near-complete kinase inactivation, we describe another kinase-compromised allele (*D149A*) offering promising new tools for CK1 research [94, 95]. Rescue capacity of human CK1ϵ is comparable to the yeast WT Hrr25 control. Furthermore, we noticed that combined removal of autophosphorylation sites in CK1ϵ also trigger Elongator inactivity, corroborating our observations for yeast Hrr25. Partial gene complementation between higher eukaryotes (i.e. plant and human cells) and yeast is proven possible and in the case of shuffles that studied conservation of Elongator or related U_34_ thiolation [96–98], it has been noted that phylogenetic barriers can be resolved by including species-specific regulator proteins that have coevolved with the pathway studied. Transferred to the gene shuffles presented here, it will be intriguing to study whether the capacity of CK1ϵ to substitute Hrr25 with regard to Elongator dependent tRNA modification can be furthered by co-expressing the mammalian homolog of Kti12 [99], which is a tRNA binder and crucial for Elongator activity in multiple species [45, 46, 54, 100].

Foremost, we would like to propose a working model on how the CK1 activity of Hrr25 influences the Elongator pathway for tRNA modification *in vivo* (Fig. 7). Hrr25 is able to undergo autophosphorylation in *cis* and *trans*, and we demonstrated that *cis*-autophosphorylation promotes dissociation of a complex formed between Hrr25 and the Sit4 phosphatase (Fig 7, top right). While Hrr25 was previously found to coprecipitate with Sit4 *in vivo* [20, 47, 48], it was unclear whether their interaction is direct or influenced by autophosphorylation of Hrr25. Our results confirm the formation of direct and stable complex between Hrr25 and Sit4, but in the absence of experimental structural data, we are unable to precisely locate the binding interface. Due to technical limitations we are also not able to determine the sequence of autophosphorylation events in Hrr25 that abrogate Sit4 binding. The inability of pHrr25 to bind to Sit4 might be caused by the fact that the free fraction of Hrr25 auto-phosphorylates itself in *cis* once it has dissociated from Sit4, which subsequently prevents it from rebinding to Sit4. We noticed that autophosphorylation patterns between FL kinase and the truncation (Hrr25 1–394) lacking the P/Q-rich C-terminus do vary. Presumably, these differences shed further light onto a regulatory role played by the P/Q-rich region for kinase performance and may explain previous findings that C-terminal autophosphorylation in mammalian CK1 can inhibit substrate phosphorylation through transient masking of the kinase domain [101, 102]. Irrespective of the precise autophosphorylation outcome, we demonstrate that recombinant Hrr25 phosphorylates a number of previously described physiological targets, namely the Elp123 subcomplex and autophagy receptors Atg19 and Atg34. Despite the limitation of *in vitro* studies, our data are in support of previous studies *in vivo* that identified a pattern of Atg19 p-sites similar to ours [22, 103, 104]. As for Elp123, we were able to pinpoint Hrr25 binding to the pocket between two WD40 domains in Elp1 and the Elp3 catalytic subunit, where we mapped multiple Hrr25 p-sites (Fig. 2D and E). Excessive Elp123 phosphorylation *in vitro* here again may not necessarily reflect a physiological state, but the majority of p-sites, which mapped outside this pocket, corresponds with previous Elongator phosphorylation studies in yeast and human cells [35, 105–108]. More importantly, multiple phosphorylation close to the tRNA binding region in Elp1 have been shown to impact on Elongator’s U_34_ modification activity [35, 109].

As judged from gene shuffling, we show that expression of human ortholog CK1ϵ (and not CK1δ), rescues phenotypes of yeast *hrr25* kinase mutants *in vivo*. Importantly, the ability of CK1ϵ to replace yeast Hrr25 function depends on the integrity of the kinase domain in CK1ϵ showing that phosphorylation activity is key to cross-species complementation. That CK1ϵ can replace Hrr25 function, strongly suggests conservation between the activity of each kinase in signalling to the tRNA modification pathway, which by itself is known to be highly conserved throughout evolution [61, 96]. Similarly, Hrr25 and CK1ϵ share conserved sites for autophosphorylation and we reveal that they are functionally relevant. As only 2%–6% of p-sites are highly conserved across species, such conserved p-sites most likely locate at strategically important parts of the kinase [110, 111]. Our finding that autophosphorylation confers to Hrr25 thermostability *in vitro* may partially explain reduced protein levels in case of quadruple/quintuple phosphoablative (*S143A/T144A/S181A/S191A/S198A*) or catalytically inactive (*K38R*) mutants (Fig. 6F and Supplementary Fig. S4B). Interestingly, protein level is much less affected in case of the *T27A/S31A* mutant (Fig. 6F), where Thr27 and Ser31 are spatially closest to the catalytic site making them plausible candidates for *cis*-autophosphorylation. They locate at the interface of Hrr25 and Sit4 in the AlphaFold model (Fig. 6E), which corroborates a potential effect of *cis*-autophosphorylation on Hrr25–Sit4 interaction.

The influence of CK1 autophosphorylation on substrate selection and effector pathway modulation still craves for thorough analysis. For instance, Hrr25 was recently implicated in CWI maintenance [112]. This role may be linked to the Elongator pathway, as our data show that quadruple/quintuple p-site ablation mutants in Hrr25 are sensitive to the CWI indicator drug CFW (Supplementary Fig. S8C), a phenotype in common between *elp3*Δ and *pin4*Δ mutants. With the latter lacking the Hrr25 phosphorylation target Pin4 involved in CWI and able to suppress the growth defect of secretion mutants (*sec12-4*, *sec2-9*) in a manner like *hrr25* kinase-dead mutants and Elongator-minus cells [28, 113], it will be interesting to study whether Hrr25 autophosphorylation may coordinate CK1 signalling into both CWI and Elongator pathways.

CK1 members, in particular, CK1ϵ and *Drosophila double-time* have long been known to be part of the clock network that drives circadian rhythms [5–8, 114]. Although a *bona fide* biological clock in yeast has been moot, *S. cerevisiae* cells maintain metabolic cycles with features of circadian rhythms. As shown by CK1ϵ inhibitors to alter metabolic cycle frequencies in analogy to prolonged clock rhythms of *double-time* mutants [115, 116] it appears that Hrr25 may also be involved in oscillations. Having shown that a major invariant out-put between Hrr25 and CK1ϵ lies with regulation of Elongator function, it will be important to ask whether the tRNA modification pathway has its stake in metabolic cycles or circadian rhythms in lower and higher eukaryotes, respectively. Our work advances previous reports showing that rather than being constitutively formed, high-fidelity modification of tRNA anticodons is subject to regulation including Elongator phosphorylation by and autophosphorylation of the CK1 isozymes Hrr25 or CK1ϵ [35, 105, 107]. In light of studies showing that tRNA modifications at U_34_ can oscillate in the cell cycle and under stress [117–119], our data indeed support the view that Elongator is dynamic and able to respond to endogenous or environmental cues in order to ensure tRNA functioning at the right time and demand of the cell.

## Supplementary Material

gkaf881_Supplemental_File

## Data Availability

All data are incorporated into the article and its online Supplementary Material. The mass spectrometry proteomics data have been deposited to the ProteomeXchange Consortium via the MassIVE partner repository with the dataset identifier PXD054138. Biological resources for this study are accessible from the corresponding authors on reasonable request.

## Supplementary data

Supplementary data is available at NAR online.
