# Supplementary material for: Autophosphorylation of conserved yeast and human casein kinase 1 isozymes regulates Elongator-dependent tRNA modifications
Source: Nucleic Acids Res. 2025 Sep 10;53(17):gkaf881. doi: 10.1093/nar/gkaf881 (PMC12421383; doi:10.1093/nar/gkaf881)
Supplement: gkaf881_Supplemental_File [file gkaf881_supplemental_file.pdf]

Supplementary Materials

Autophosphorylation of conserved yeast and human casein kinase 1 isozymes regulates Elongator-dependent tRNA modifications

Maria Friederike Landrock[1]†, Rościsław Krutyhołowa[2,3]†, Pauline Böhnert[1], Jarosław Mazur[2,4], Małgorzata Honc[2], Alexander Hammermeister[1,2], Larissa Bessler[5], David Scherf[1], Anna Elms[1], Natalia Radczuk[2], Bozena Skupien-Rabian[2], Urszula Jankowska[2], Friedrich W. Herberg[6], Mark Helm[5], Roland Klassen[1], Sebastian Glatt[2,7]*, and Raffael Schaffrath[1]*

[1] Department of Microbiology, Institute of Biology, University of Kassel, 34132 Kassel, Germany
[2] Małopolska Centre of Biotechnology, Jagiellonian University, 30387 Krakow, Poland
[3] Institute of Molecular Biology and Biophysics, ETH Zurich, 8093 Zurich, Switzerland
[4] Institute of Medical Microbiology, University of Zurich, 8006 Zurich, Switzerland
[5] Institute of Pharmaceutical and Biomedical Sciences, Johannes Gutenberg University of Mainz, 55128 Mainz, Germany
[6] Institute of Biology, Department of Biochemistry, University of Kassel, 34132 Kassel, Germany
[7] Department for Biological Sciences and Pathobiology, University of Veterinary Medicine Vienna, 1210 Vienna, Austria

* To whom correspondence should be addressed. Tel: +49 561 804-4175; Fax: +49 561 804-4337; Email: schaffrath@uni-kassel.de. Correspondence may also be addressed to Sebastian Glatt. Email: Sebastian.Glatt@vetmeduni.ac.at

† The first two authors should be regarded as Joint First Authors.

1. Supplementary Tables

Table S1. Yeast strains used and generated in this study.

Strain	Genotype	Source
Saccharomyces cerevisiae		
BY4741	*MAT*a *his3Δ1 leu2Δ0 met15Δ0 ura3Δ0*	Euroscarf *
Y03160	BY4741; *chs3Δ::KanMX4*	Euroscarf
UMY2893	*MATα SUP4 leu2-3,112 trp1-1 can1-100 ura3-1 ade2-1 his3-11,15*	(1)
YAH072	UMY2893; *ELP1-(c-myc)$_3$::SpHIS5 KTI12-(HA)$_6$::KlTRP1 hrr25-E52D::loxP*	This study
YAH108	UMY2893; *ELP1-(c-myc)3::SpHIS5 KTI12-(HA)6::KlTRP1*	This study
YML001	UMY2893; *ELP1-(c-myc)$_3$::KlTRP1 SIT4-HA::loxP KTI12-(His)$_6$::loxP*	This study
YML043	as YML001, but *hrr25Δ::SpHIS5* + [pCM12.2 (*HRR25*)]	This study
YML080	as YAH108, but *elp3Δ::loxP*	This study
YML088	as YAH108, but *hrr25-K38A::loxP*	This study
YML089	as YAH108, but *hrr25-K38R::loxP*	This study
YML090	as YAH108, but *hrr25-E52D::loxP*	This study
YML091	as YAH108, but *hrr25-D149A::loxP*	This study
YML092	as YAH108, but *hrr25-Q395STOP::loxP*	This study
Kluyveromyces lactis		
AWJ137	*MATa leu2 trp1* [pGKL1+ pGKL2+]	(2)

Notification: Some of the strains have been used as recipient for plasmid transformation and/or 5-FOA chase out.
* http://www.euroscarf.de/index.php?name=News

Table S2. Primers used in this study.

Name	Sequence (5′ → 3′)	Usage **
S3_ELP1_Fwd	TACCTGTTCCAGAGATTCATGATTTCCCTAAGAGTCATATTGTTGATTT TCGTACGCTGCAGGTCGAC	tag
S2_ELP1_Rev	CTTTACGAGCACTATAGACAGTAATTTATATAACTAAGAAAATGGTAT GCATCGATGAATTCGAGCTCG	tag
S3_Hs-CSNK1E_6xHA_Fwd	GCCATTTGACCATCTCGGGAAGCGTACGCTGCAGGTCGAC	tag
S3_Hs-CSNK1-D1_6xHA_Fwd	CTTCAGTCTGTCGTGCACCGACGTACGCTGCAGGTCGAC	tag
S3_Hs-CSNK1-D2_6xHA_Fwd	CCTTTCGAACACCACGGCAAGCGTACGCTGCAGGTCGAC	tag
S3_Hs-CSNK1-D3_6xHA_Fwd	CAGCGCCCACGACGCACCTACCGTACGCTGCAGGTCGAC	tag
S3_Hs-CSNK1-DX1_6xHA_Fwd	GTGTGGCGTCTTGGTTGCAGACGTACGCTGCAGGTCGAC	tag
6xHA-in-YCplac-Cterm_Rev	CCAGTGTGATGGATATCTGCAGAATTCTTAGCTAGAAGCGTAATCTGGAA	tag
Elp3koF	AGTCCTAAAAGCACCTAAGGAAAATCGAAGAACACCCTGACAAAGCAGC TGAAGCTTCGTACGC	ko
Elp3koR	AAAACCGGCCATGTCGGCGGCACATAAAAGTTCTATTTACCTTTAGCATA GGCCACTAGTGGATCTG	ko
Hrr25_KO_Fwd	AAAAACCAAAAAGAAAAGATATATTTATAGAAAGGATACATTAAAAAGA GCAGCTGAAGCTTCGTACGC	ko
Hrr25_KO_Rev	TCTATATATACATATGTTTATTTTTGTGCGTTTTGAGCAATATATGTTGCGC ATAGGCCACTAGTGGATCTG	ko
Hrr25_3'-LEU_Fw	GCAACATATATTGCTCAAAACGCACAAAAATAAACATATGTATATATAGA GCATAGGCCACTAGTGGATCTG	sdm

Name	Sequence (5′ → 3′)	Usage **
Hrr25_3'-LEU_Rv	GTATATGTAAATAATAATAATAATATATATATATATATGTGTGTGTGTATGCAGCTGAAGCTTCGTACGC	sdm
Hrr25_3'UTR_Rev	GTGATTGATCTTCGATCGTTAGC	sdm
Hrr25_Mutation_Rev_NEW	GGTGCTCCGCTCCCCCTTAATTTCCTTCCCGAAGAAAAAAATGCATAAAAAAAG	sdm
Hrr25_T27A_Fwd	ATTTACCACGGCGCTAACTTAATTAGTGGTGAAGAAGTAG	sdm
Hrr25_T27A_Rev	AATTAAGTTAGCGCCGTGGTAAATGTCACCAAA	sdm
Hrr25_T27A_S31A_Fwd	ATTTACCACGGCGCTAACTTAATTGCTGGTGAAGAAGTAGCCATCAAG	sdm
Hrr25_T27A_S31A_Rev	ACTTCTTCACCAGCAATTAAGTTAGCGCCGTGGTAAATGTCACCAAA	sdm
Hrr25_K38A_Fwd	GGTGAAGAAGTAGCCATCGCTCTGGAATCGATCAGGTCC	sdm
Hrr25_K38A_Rev	GGACCTGATCGATTCCAGAGCGATGGCTACTTCTTCACC	sdm
Hrr25_K38R_Fwd_NEW	CCATCAGGCTGGAATCGATCAGGTCCA	sdm
Hrr25_K38R_Rev	CTGGACCTGATCGATTCCAGCCTGATGGCTACTTCTTCACCAC	sdm
Hrr25_E52D_Fw	CCTCAATTGGACTATGACTCCCGCGTCTACAG	sdm
Hrr25_E52D_Fwd (Krakow)	TTGGACTATGACTCCCGCGTCTACAGATACTTAA	sdm
Hrr25_E52D_Rev	AGACGCGGGAGTCATAGTCCAATTGAGGATGTCT	sdm
Hrr25_S143A_Fwd	GTAGGACGCCGTGGTGCCACCGTTCATGTTAT	sdm
Hrr25_S143A_Rev	ATAACATGAACGGTGGCACCACGGCGTCCTAC	sdm
Hrr25_T144A_Fwd	GGACGCCGTGGTAGCGCCGTTCATGTTATTG	sdm
Hrr25_T144A_Rev	CAATAACATGAACGGCGCTACCACGGCGTCC	sdm
Hrr25_T144A-incl-S143A_Fwd	CGTGGTGCCGCCGTTCATGTTATTGATTTCGGT	sdm
Hrr25_T144A-incl-S143A_Rev	ACATGAACGGCGGCACCACGGCGTCCTA	sdm
Hrr25_D149A_Fwd	GTTATTGCTTTCGGTCTATCAAAGAAATACCGA	sdm
Hrr25_D149A_Rev	CCGAAAGCAATAACATGAACGGTGCTACCAC	sdm
Hrr25_S181A_Fwd	CGTTATGCAGCTGTCAATACGCATCTTGGAATAGA	sdm
Hrr25_S181A_Rev	ATTGACAGCTGCATAACGAGCTGTACCTGT	sdm
Hrr25_S191A_Fwd	AGAGCAAGCTAGAAGAGATGACTTAGAATCACTA	sdm
Hrr25_S191A_Rev	CTCTTCTAGCTTGCTCTATTCCAAGATGCGTATT	sdm
Hrr25_S198A_Fwd	ACTTAGAAGCACTAGGTTATGTCTTGATCTATTTT	sdm
Hrr25_S198A-incl-S191A_Rev	ACCTAGTGCTTCTAAGTCATCTCTTCTAGCTTG	sdm
Hrr25_Q395STOP_Fwd NEW	TTGAGATAACAGCAGCCGCAGCAGC	sdm
Hs-CSNK1-D1_Fwd	TCACAGATTCCTGGTCGGGTGGCTTCCAGTGGTCTTCAGTCTGTCGTGCACCGATGAGAATTCTGCAGATATCCATCACA	sdm
Hs-CSNK1-D1_Rev	CAGAATTCTCATCGGTGCACGACAGACTGAAGACCACTGGAAGCCACCCGACCAGGAATCTGTGAGGTGGACATGCGA	sdm
HsCSNK1D_K38R_Fwd	CCATCAGACTTGAATGTGTCAAAACCAAACAC	sdm
HsCSNK1D_K38R_Rev	TTCAAGTCTGATGGCAACCTCTTCTCCTG	sdm
HsCSNK1-X1_part1_Fwd	CCTCACAGGTAGGTGCAGAGCTCCCGGGCGGGCGCTGGGCCTCGGGCTACCCCGTCCGAGTCGAATTCTGCAGATATCCATCACA	sdm
HsCSNK1-X1_part1_Rev	CAGAATTCGACTCGGACGGGGTAGCCCGAGGCCCAGCGCCCGCCCGGGAGCTCTGCACCTACCTGTGAGGTGGACATGCGA	sdm
HsCSNK1-X1_part2_Fwd	CGAGTCAAGAGCGGGCTGTGGTTTCTCCAGGGCTTCTTCTTTCCTTTCTTCCCCCCATTTTTGAGAGTGAATTCTGCAGATATCCATCACA	sdm
HsCSNK1-X1_part2_Rev	CAGAATTCACTCTCAAAAATGGGGGGAAGAAAGGAAAGAAGAAGCCCTGGAGAAACCACAGCCCGCTCTTGACTCGGACGGGGTAGC	sdm
HsCSNK1-X1_part3_Fwd	GAGAGTTTTTAATCTCGCCAGGGTGTGGCGTCTTGGTTGCAGATGAGAATTCTGCAGATATCCATCACA	sdm
HsCSNK1-X1_part3_Rev	CAGAATTCTCATCTGCAACCAAGACGCCACACCCTGGCGAGATTAAAAACTCTCAAAAATGGGGGGAAG	sdm
Hs-CSNK1E_S31A_Fwd	CATCGCCGCTGGTGAGGAAGTCGCCAT	sdm
Hs-CSNK1E_S31A_Rev	CTCACCAGCGGCGATGTTGGCACCCAG	sdm
Hs-CSNK1E_K38R_Fwd	GTCGCCATCAGACTGGAGTGTGTGAAGACAAAG	sdm
Hs-CSNK1E_K38R_Rev	CACTCCAGTCTGATGGCGACTTCCTCACC	sdm
HsCSNK1E_S181A_Fwd	GCTACGCTGCCATCAACACGCACCTGG	sdm

Name	Sequence (5′ → 3′)	Usage **
HsCSNK1E_S181A_Rev	CGTGTTGATGGCAGCGTAGCGGGCCGTG	sdm
HsCSNK1E_S191A_Fwd	GAGCAAGCCCGTCGAGATGACCTGGA	sdm
HsCSNK1E_S191A_Rev	CTCGACGGGCTTGCTCAATGCCCAGGTGC	sdm
HsCSNK1E_S198A_Fwd	CCTGGAGGCCCTGGGCTACGTGCTCATG	sdm
HsCSNK1E_S198A_Rev	CGTAGCCCAGGGCCTCCAGGTCATCTCGACGG	sdm
Hrr25_Exchange_Fwd	AAGATATATTTATAGAAAGGATACATTAAAAAGAGATGGACTTAAGAGT AGGAAGGAAA	oep
Hrr25_FL_Exchange_ Rev	TGCGAACTTTGGACCTGCTGCTGCGGCTGCTGTTGTCTCAAGTTTTCC ATACCTTTATCTA	oep
Hrr25_TR_Exchange_ Rev	TGCGAACTTTGGACCTGCTGCTGCGGCTGCTGTTATCTCAAGTTTTCC ATACCTTTA	oep
Hrr25_Exchange-in-YCplac33_Fw	CAGCTATGACCATGATTACGCCAAGCTTGCATGGATCTAAACCAATTC GGTATTGTC	oep
Hrr25_Exchange-in-YCplac33_Re	GTGAATTCGAGCTCGGTACCCGGGGATCCTCTAGGAGCCCTTTCTGC AACGAATT	oep
Hs-CSNK1D_Exchange-in-YCplac_Fwd	CCAAGCATACAATCAACTATCTCATATCATATGGAGCTGAGAGTCGGG AAC	oep
Hs-CSNK1D-1_ Exchange-in-YCplac_ Rev	CGGCCGCCAGTGTGATGGATATCTGCAGAATTCTCATCGGTGCACGA CAGAC	oep
Hs-CSNK1D-2_ Exchange-in-YCplac_ Rev	CGGCCGCCAGTGTGATGGATATCTGCAGAATTCCTACTTGCCGTGGT GTTCG	oep
Hs-CSNK1D-3_ Exchange-in-YCplac_ Rev	CGGCCGCCAGTGTGATGGATATCTGCAGAATTCTCAGTAGGTGCGTC GTGGG	oep
Hs-CSNK1-X1_ Exchange-in-Ycplac_Rev	CGGCCGCCAGTGTGATGGATATCTGCAGAATTCTCATCTGCAACCAA GACGCC	oep
Hs-CSNK1E_Exchange-in-YCplac_Fwd	CCAAGCATACAATCAACTATCTCATATCATATGGAGCTACGTGTGGGG AAC	oep
Hs-CSNK1E_Exchange-in-YCplac_Rev	CGGCCGCCAGTGTGATGGATATCTGCAGAATTCTCACTTCCCGAGAT GGTCA	oep
N_Elp1_Fw	GTAAGCGTGCTCGTGGTAAG	v
N_Elp1_Rv	CCAAATGGCGCTGTTATTGG	v
ELP3FW	CGATAAGACAGTGAGAGAAGG	v
Elp3_Seq-1_Fwd	CGTTGTCCTCACATTGCATATAC	v
Elp3_Tag_Check_Rev	GCAGTTACTCCCATCATAAAAGG	v
Hrr25_Control_3'-LEU_ Rv	GGTAAGAAGGCCAGAATTGTA	v
Hrr25_Mut_Control_Fw	CTTCTTCCCGTCTGTGTATTTAC	v
Hrr25_Mutation_Rev_ NEW	GGTGCTCCGCTCCCCCTTAATTTCCTTCCCGAAGAAAAAAATGCATAA AAAAAG	v
Hrr25_rtPCR-1_Fwd	GCGTGGAAACTCTATGTTCAGG	v
Hrr25_rtPCR-1_Rev	TTCTCCACCATCGCCTTTGT	v
Hrr25_Seq_N-Term_Rev	CATTATATTCACCCTCTCTG	v
Hrr25_Seq_C-Term_Fwd	GTTACTACAGCAGCAACAAA	v
Hrr25_Seq_1_Fwd	CTTAGTAGCATCTTTAAACC	v
Hrr25_Seq_2_Rev	CCTTTGTGTAACGCAACATTGT	v
Hrr25_Seq_3_Rev	CCAAATTGACTGGCCAGCTG	v
Hrr25_Seq_4_Fwd	CTAGGCCCATCTTTGGAAGA	v
Hrr25_Seq_5_Fwd	GGCAAGGCTGTTTAAAGATC	v
Hrr25_3'UTR_Rev	GTGATTGATCTTCGATCGTTAGC	v

Name	Sequence (5′ → 3′)	Usage **
HsCSNK1D_Seq-1_Fwd	GACGACAAGCCTGACTACTC	v
Hs-CSNK1E_rtPCR-1_Fwd	GAGCAAAGCCGTCGAGATGA	v
Hs-CSNK1E_rtPCR-1_Rev	CGGAGGGATAGCCTTTGCAG	v
KlLEU2_qPCR_Fwd	TCTGGAAGAGGCAAGCACG	v
KlLEU2_qPCR_Rev	GTGACGGAGTTGCTTGGGA	v
M13_FW_-40	GGTTTTCCCAGTCACGAC	v
M13_RV_-27	GGAAACAGCTATGACCATG	v
N_UBA4/MOCS3_RV	TACATGCGTACACGCGTCTG	v
P-ADH1-Seq_Fwd	GCTATCAAGTATAAATAGACCTGC	v
pUG73/KlLEU rv	CACCTAGTAAGACAGCATCG	v
T7	TACGACTCACTATAGGGGAATTG	v
T7ter	GCTAGTTATTGCTCAGCGGTG	v

****** Abbreviations used:
(i) tag – epitope tagging (ii) ko – gene knock-out; (iii) sdm – site-directed mutagenesis; (iv) oep – overlap extension PCR; (v) v – verification PCR

Table S3. Plasmids used and generated in this study.

Plasmid	Genotype	Source
YCplac33	*Amp^R CEN4 ARS1 ScURA3*	(3)
YCplac111	*Amp^R CEN4 ARS1 ScLEU2*	(3)
pCM12.2	YCplac33; P_{native}-*ScHRR25*-T_{none}	(4)
pML004	YCplac111; P_{native}-*ScHRR25*-T_{native}	This study
pML008	YCplac111; P_{ADH1}-*HsCSNK1E*-T_{CYC1}	This study
pML052	YCplac111; P_{native}-*Schrr25-T27A/S31A*-T_{native}	This study
pML054	YCplac111; P_{native}-*Schrr25-K38A*-T_{native}	This study
pML055	YCplac111; P_{native}-*Schrr25-K38R*-T_{native}	This study
pML057	YCplac111; P_{native}-*Schrr25-E52D*-T_{native}	This study
pML062	YCplac111; P_{native}-*Schrr25-S143A/S181A/S191A/S198A*-T_{native}	This study
pML063	YCplac111; P_{native}-*Schrr25-S143A/T144A/S181A/S191A/S198A*-T_{native}	This study
pML065	YCplac111; P_{native}-*Schrr25-T144A/S181A/S191A/S198A*-T_{native}	This study
pML066	YCplac111; P_{native}-*Schrr25-D149A*-T_{native}	This study
pML071	YCplac111; P_{native}-*Schrr25-S181A/S191A/S198A*-T_{native}	This study
pML085	YCplac111; P_{native}-*Schrr25-Q395STOP*-T_{native}	This study
pML102	YCplac111; P_{ADH1}-*HsCSNK1E-K38R*-T_{CYC1}	This study
pML104	YCplac111; P_{ADH1}-*HsCSNK1E-S181A*-T_{CYC1}	This study
pML105	YCplac111; P_{ADH1}-*HsCSNK1E-S181A/S191A/S198A*-T_{CYC1}	This study
pML106	YCplac111; P_{ADH1}-*HsCSNK1E-S191A*-T_{CYC1}	This study
pML108	YCplac111; P_{ADH1}-*HsCSNK1E-S198A*-T_{CYC1}	This study
pML110	YCplac111; P_{ADH1}-*HsCSNK1E*-$(HA)_6$-T_{CYC1}	This study
pML111	YCplac111; P_{ADH1}-*HsCSNK1E-K38R*-$(HA)_6$-T_{CYC1}	This study
pML117	YCplac111; P_{ADH1}-*HsCSNK1D1*-T_{CYC1}	This study
pML120	YCplac111; P_{ADH1}-*HsCSNK1D1*-$(HA)_6$-T_{CYC1}	This study
pML127	YCplac111; P_{ADH1}-*HsCSNK1D2*-T_{CYC1}	This study
pML130	YCplac111; P_{ADH1}-*HsCSNK1D2*-$(HA)_6$-T_{CYC1}	This study
pML137	YCplac111; P_{ADH1}-*HsCSNK1D3*-T_{CYC1}	This study
pML140	YCplac111; P_{ADH1}-*HsCSNK1D3*-$(HA)_6$-T_{CYC1}	This study
pML149	YCplac111; P_{ADH1}-*HsCSNK1X1*-T_{CYC1}	This study
pML152	YCplac111; P_{ADH1}-*HsCSNK1X1*-$(HA)_6$-T_{CYC1}	This study
pML160	YCplac111; P_{ADH1}-*HsCSNK1E-S31A*-T_{CYC1}	This study

2. Supplementary Figures

Figure S1:

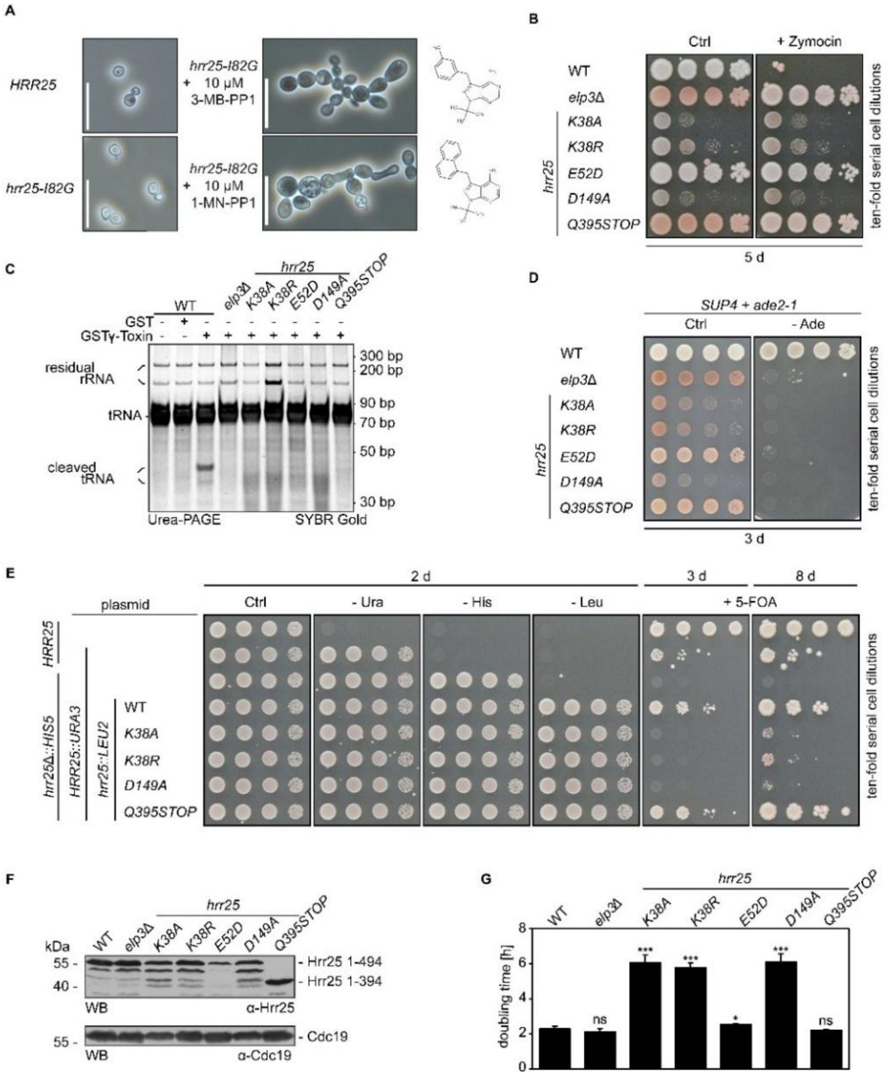

Fig. S1. Hrr25 is important for viability and growth of *S. cerevisiae* cells. (A) Chemical and genetic inhibition of Hrr25 results in aberrant cell morphologies. The gatekeeper mutant *hrr25-I82G* (5) allows for the chemical inhibition by ATP analogue 3-MB-PP1 and 1-MN-PP1 (scale bar = 20 µm). (B) *hrr25* mutants confer zymocin resistance. Serial dilutions of the indicated strains were replica spotted on YPD plates (control, Ctrl), or supplemented with 100 % (v/v) zymocin. (C) *hrr25* mutants resist cleavage by γ-toxin. Bulk tRNA from *hrr25* mutants was purified and subjected to γ-toxin cleavage. tRNA halves were separated by urea-PAGE and stained with SYBR^{TM}-Gold. (D) *hrr25* mutants lack *SUP4* read-through. *SUP4* assay with read-through of *ade2-1* mutation, strains were replica spotted on YNB plates containing (control, Ctrl) or lacking adenine (-Ade). Cultivation was for 4-5 days at 30 °C. (E) Catalytically active Hrr25 is essential for viability of yeast cells. Serial dilutions of the indicated strains were replica spotted on YNB plates (Ctrl, control), plates lacking a constituent, (Ura, uracil; His, histidine; Leu, leucine) or supplemented with 5-FOA (for assay details, see Fig. 1). Cultivation was for 3-8 days at 30 °C. (F) Protein expression levels of *hrr25* mutants. Proteins were isolated from indicated strains at mid-exponential phase and subjected to Western blot analysis. Immunodetection of Hrr25 was achieved utilizing anti-Hrr25 antibodies. Equal loading was confirmed by detection of Cdc19. (G) *hrr25*-kinase-dead mutations lead to retarded growth. Doubling time analysis was conducted in an end point approach. The statistical significance of four independent results was tested with a one-way t-test (***P < 0.001, *P < 0.05, ns > 0.05; ns, not significant).

Figure S2:

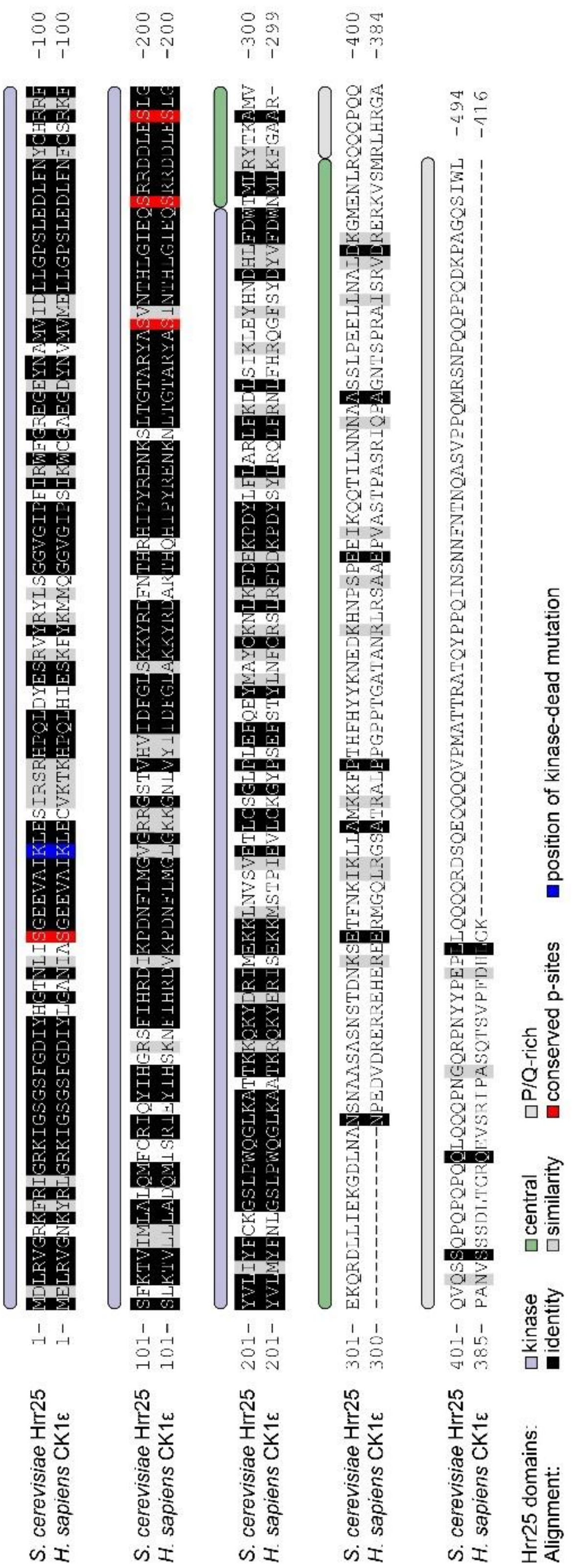

Fig. S2. Alignment of Hrr25 and *Hs*CK1ε. Hrr25 domains are indicated in purple (kinase domain), green (central domain) and grey (P/Q-rich domain). Protein sequences of Hrr25 from *S. cerevisiae* and CK1ε from *H. sapiens* were aligned using AlignX. Amino acid identity and similarity are highlighted in black and grey, respectively. Position of a kinase dead mutation is shown in blue (K38). Red highlights autophosphorylation sites of Hrr25 that have corresponding residues in CK1ε (S31, S181, S191, S198).

Figure S3:

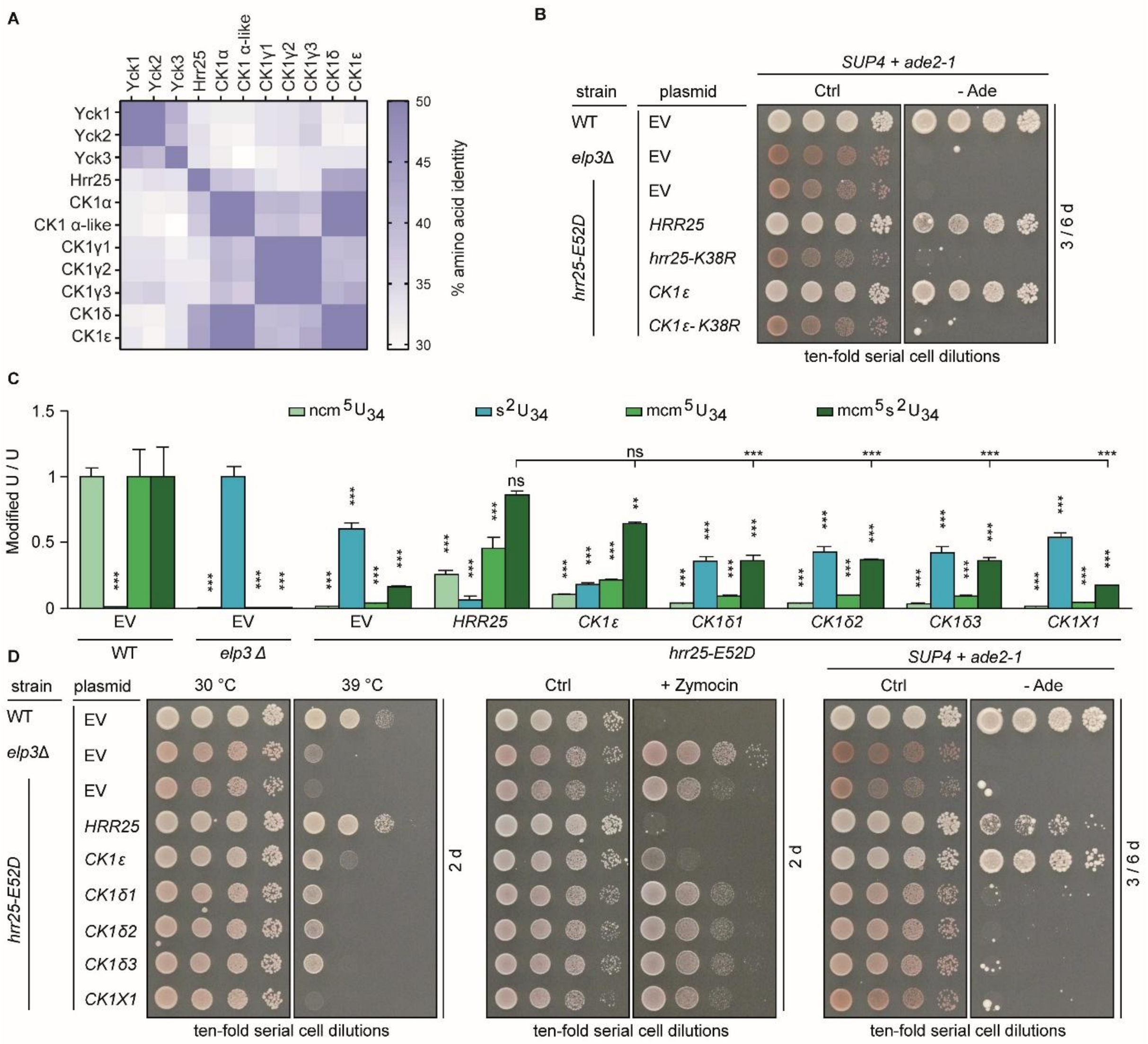

Fig. S3. CK1ε is the human homolog of Hrr25. (A) Heatmap representation of pairwise sequence identity of all human and yeast casein kinases. Hrr25 is more similar to CK1δ and CK1ε than to yeast Yck1, Yck2 and Yck3. (B) Loss of *SUP4* read-through caused by *HRR25* mutation can be reversed by CK1ε. Strains were replica spotted on YNB plates lacking leucine and containing (control, Ctrl) or lacking adenine (-Ade). Cultivation was for 3-6 days at 30 °C. (C) LC-MS/MS analysis of U_{34} modification shows that CK1δ1, CK1δ2, CK1δ3 and CK1X1 failed to rescue tRNA modification defects of *hrr25-E52D*. For measurement details and statistical significance analysis, see Fig.1. (D) Thermosensitivity, zymocin resistance and loss of *SUP4* read-through caused by *HRR25* mutation can be reversed by CK1ε but not by CK1δ. Serial dilutions of the indicated strains were replica spotted on YNB plates lacking leucine (control, Ctrl), and/or supplemented with 100% (v/v) zymocin. For *SUP4* assay with read-through of *ade2-1* mutation, strains were replica spotted on YNB plates lacking leucine and containing (control, Ctrl) or lacking adenine (-Ade). Cultivation was for 2-6 days at 30 °C.

Figure S4:

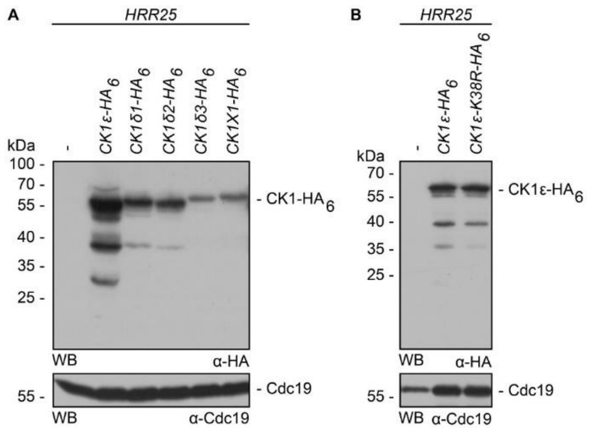

Fig. S4. Orthologous CK1 proteins can be expressed in *S. cerevisiae*. (A/B) The indicated strains were cultivated in YNB media lacking leucine and harvested at mid-exponential phase. Proteins were isolated and subjected to Western blot analysis. Immunodetection of CK1 isoenzymes was achieved utilizing anti-HA antibody, Cdc19 was used as a loading control.

Figure S5:

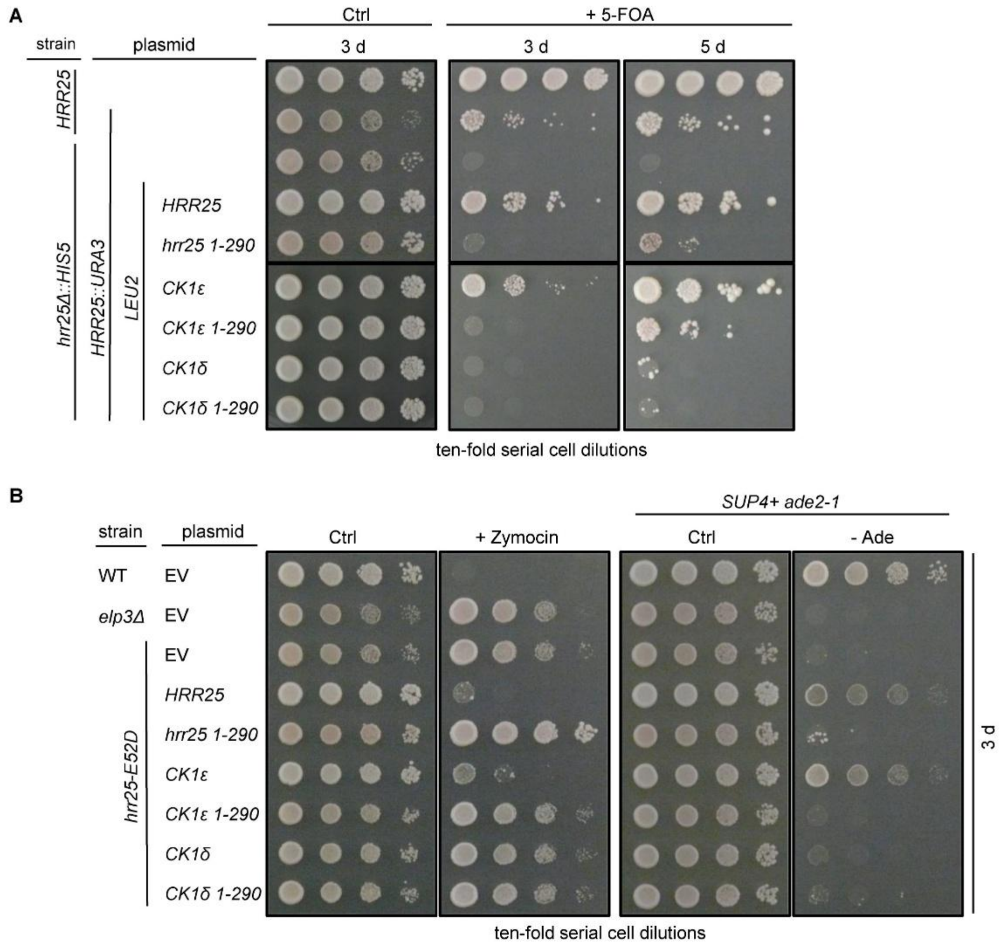

Fig. S5. Kinase domains of CK1ε and Hrr25 weakly complement *hrr25* deletion. (A) The expression of CK1ε and Hrr25 kinase domains alone partially compensates the lethal *hrr25* deletion, whereas the kinase domain of CK1δ does not. Serial dilutions of the indicated strains were replica spotted on YNB plates (Ctrl, control) or supplemented with 5-FOA (for assay details, see Fig. 1). Cultivation was for 3-5 days at 30 °C. (B) Although the kinase domains of Hrr25 and CK1ε alone can rescue the growth of *hrr25Δ*, Elongator-minus phenotypes were observed on zymocin and in the *SUP4* assay. Serial dilutions of the indicated strains were replica spotted on control YNB plates lacking leucine (Ctrl), or supplemented with 100% (v/v) zymocin. For *SUP4* assay with read-through of *ade2-1* mutation, strains were replica spotted on control YNB plates lacking leucine (Ctrl) or lacking adenine (-Ade). Cultivation was for 3 days at 30 °C.

Figure S6:

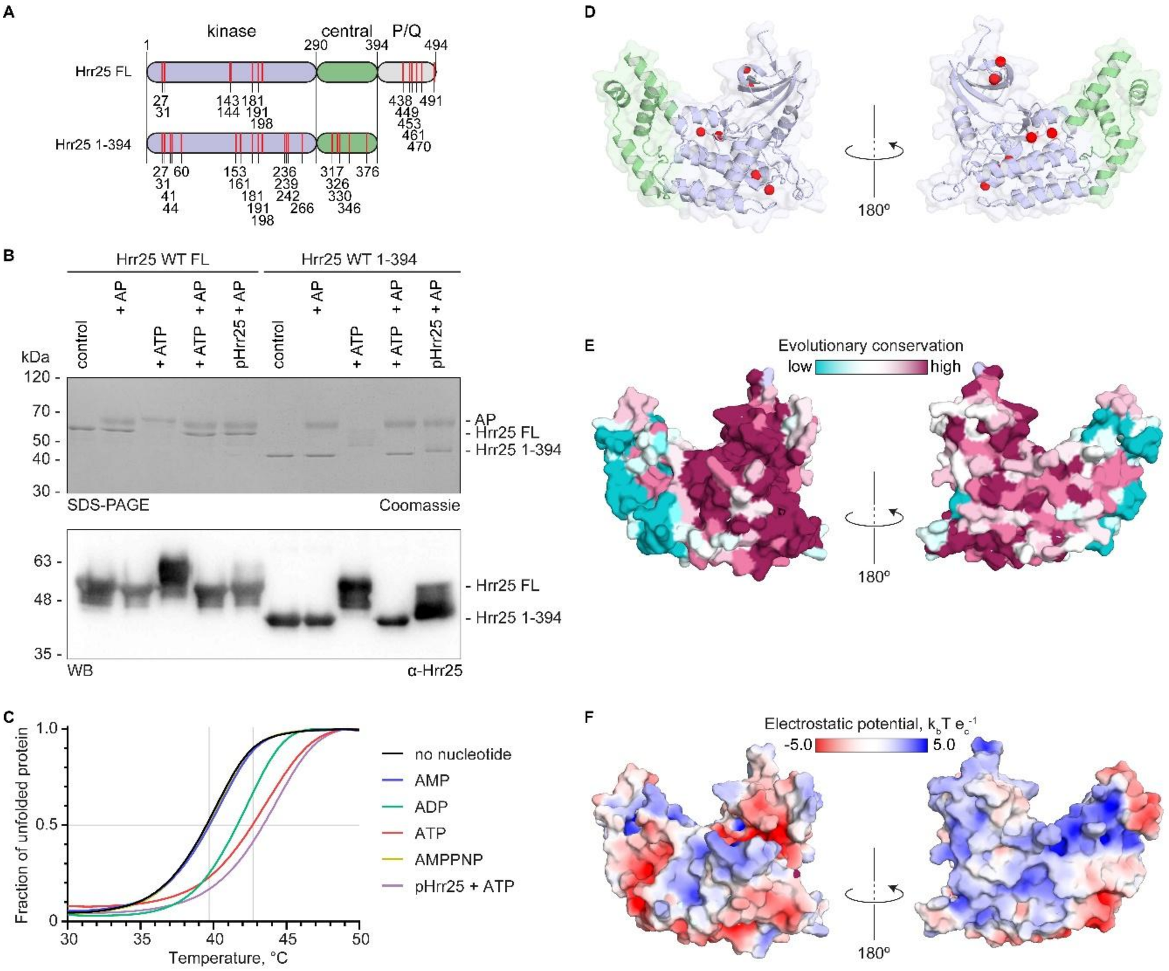

Fig. S6. Biophysical, biochemical and structural analysis of autophosphorylation of Hrr25. (A) MS analysis comparing phosphorylation patters of full-length Hrr25 (FL) and the truncated version (Hrr25 1-394). Hrr25 1-394 exhibits a much broader and more promiscuous phosphorylation profile, with numerous phosphorylation events whose biochemical significance remains uncertain. (B) Hrr25 autophosphorylation can be reversed by alkaline phosphatase treatment (AP). Both, FL and 1-394 Hrr25 exhibit an autophosphorylation-dependent shift on SDS-PAGE upon incubation for 1 hour at 30 °C. In the presence of alkaline phosphatase shifts are no longer present in all studies samples, including pre-autophosphorylated Hrr25. (C) Thermal shift assay of Hrr25 FL in presence of ATP derivatives. pHrr25 sample indicates Hrr25 FL pre-autophosphorylated for 1 hour at 30 °C. N=3, denaturation curves are scaled between 0 and 1. (D) Structural overview of Hrr25 phosphorylation sites identified in this study. (E) Surface conservation of Hrr25 assessed against the list of 1000 homologs. Conservation was evaluated using ConSurf server. (F) Calculated electrostatic surface charge distribution of Hrr25 1-394. The calculation was carried out using APBS and visualized using PyMOL.

Figure S7:

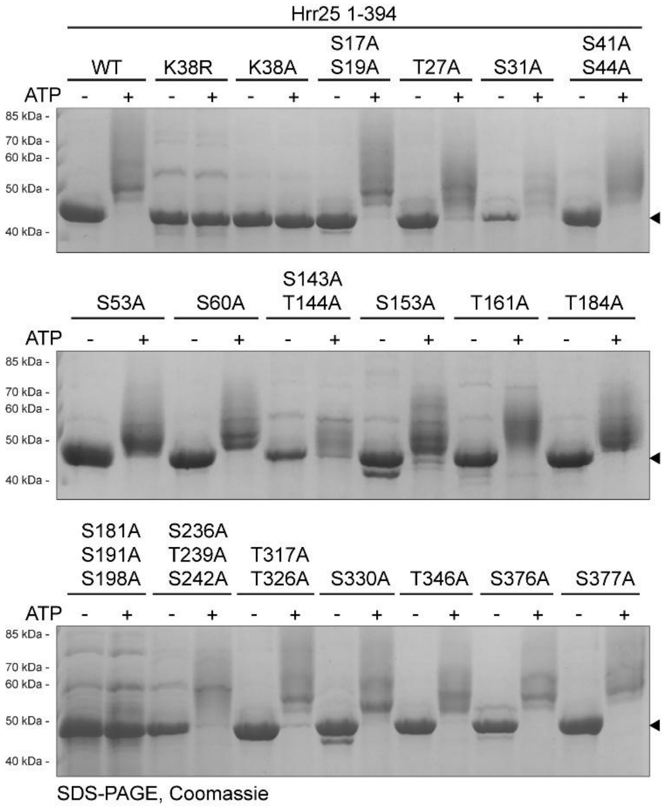

Fig. S7. Analysis of Hrr25 1-394 mutants with PhosTag. Purified proteins were incubated with and without ATP and their phosphorylation status was analysed using PhosTag containing gels. Autophosphorylation was not observed for K38R, K38A and the triple mutant S181A/S191A/S198A. All other mutants seem to undergo autophosphorylation as indicated by the shift of the Hrr25 signal (marked by the arrowhead).

Figure S8:

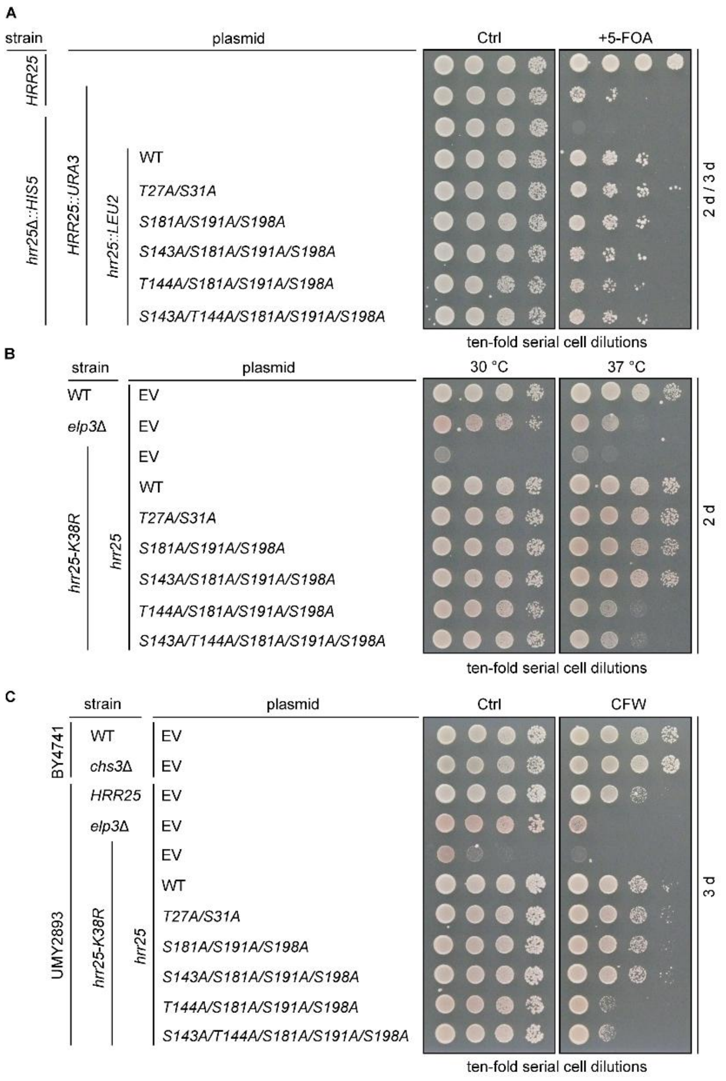

Fig. S8. Autophosphorylation of Hrr25 positively regulates its function. (A) Phosphoablative mutation of *HRR25* allele is not lethal. Serial dilutions of the indicated strains were replica spotted on YNB plates (Ctrl, control) and plates supplemented with 5-FOA. Cultivation was for 2-3 days at 30 °C. (B) Autophosphorylation of Hrr25 positively regulates thermotolerance. Serial dilutions of the indicated strains were replica spotted on YNB plates lacking leucine and cultivated for 2 days at 30 °C or 37 °C to test for thermotolerance. (C) Autophosphorylation of Hrr25 positively regulates cell wall integrity. Serial dilutions of the indicated strains were replica spotted on YNB plates lacking leucine without (Ctrl, control) or supplemented with 50 µg/ml calcofluor white (CFW). Cultivation was for 3 days at 30 °C.

Figure S9:

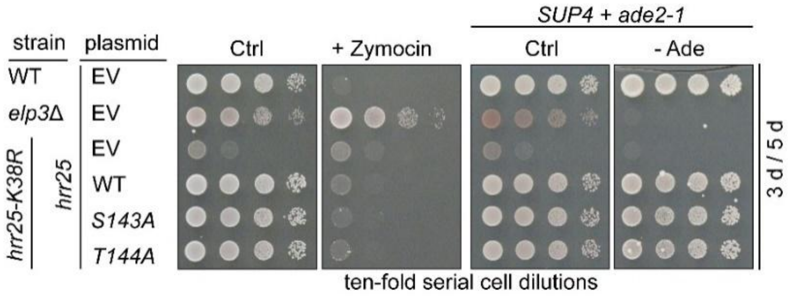

Fig. S9. Single phosphoablative substitutions of S143 and T144 result in wildtype-like phenotypes. Serial dilutions of the indicated strains were replica spotted on YNB plates lacking leucine (Ctrl, control) and plates supplemented with 100% (v/v) zymocin. For *SUP4* assay with read-through of *ade2-1* mutation, strains were replica spotted on YNB plates lacking leucine and containing (control, Ctrl) or lacking adenine (-Ade). Cultivation was for 3-5 days at 30 °C.

Figure S10:

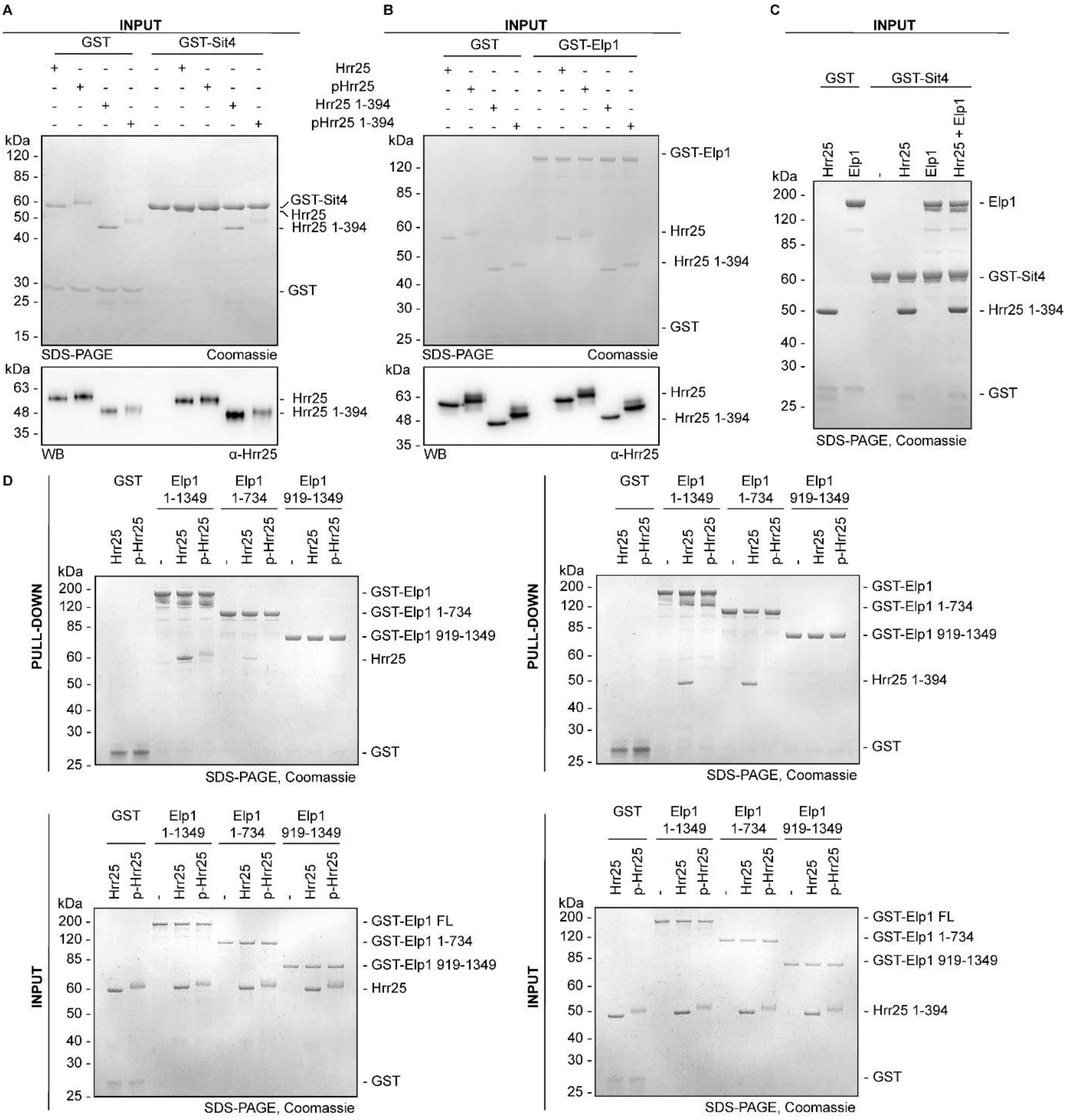

Fig. S10. Input controls of GST-pull-downs described in main text Fig. 6. (A/B/C) Input controls of GST pull-down assays described in the Fig. 6A, Fig. 6B and Fig. 6C, respectively. (D) Hrr25 binds to the N-terminal part of Elp1. Hrr25 FL and Hrr25 1-394 recognize the same region of Elp1. GST pull-down and corresponding inputs are shown, free GST was used as a specificity control.

3. Supplementary References

1. Huang, B., Johansson, M.J.O. and Byström, A.S. (2005) An early step in wobble uridine tRNA modification requires the Elongator complex. *RNA (New York, N.Y.),* 11, 424–436.
2. Kämper, J., Esser, K., Gunge, N. and Meinhardt, F. (1991) Heterologous gene expression on the linear DNA killer plasmid from Kluyveromyces lactis. *Curr Genet,* 19, 109–118.
3. Gietz, R.D. and Sugino, A. (1988) New yeast-Escherichia coli shuttle vectors constructed with in vitro mutagenized yeast genes lacking six-base pair restriction sites. *Gene,* 74, 527–534.
4. Mehlgarten, C., Jablonowski, D., Breunig, K.D., Stark, M.J.R. and Schaffrath, R. (2009) Elongator function depends on antagonistic regulation by casein kinase Hrr25 and protein phosphatase Sit4, *Molecular Microbiology.* 73, 869–881.
5. Bodenmiller, B., Wanka, S., Kraft, C., Urban, J., Campbell, D., Pedrioli, P.G., Gerrits, B., Picotti, P., Lam, H. and Vitek, O. *et al.* (2010) Phosphoproteomic analysis reveals interconnected system-wide responses to perturbations of kinases and phosphatases in yeast. *Science Signaling,* 3, rs4.
